# Supplementary material for: Entorhinal and ventromedial prefrontal cortices abstract and generalize the structure of reinforcement learning problems
Source: Neuron. 2021 Feb 17;109(4):713–723.e7. doi: 10.1016/j.neuron.2020.11.024 (PMC7889496; doi:10.1016/j.neuron.2020.11.024)
Supplement: Document S1. Figures S1–S4 and Tables S1–S3 [file mmc1.pdf]

Neuron, Volume *109*

Supplemental Information

Entorhinal and ventromedial prefrontal cortices abstract and generalize the structure of reinforcement learning problems

Alon Boaz Baram, Timothy Howard Muller, Hamed Nili, Mona Maria Garvert, and Timothy Edward John Behrens

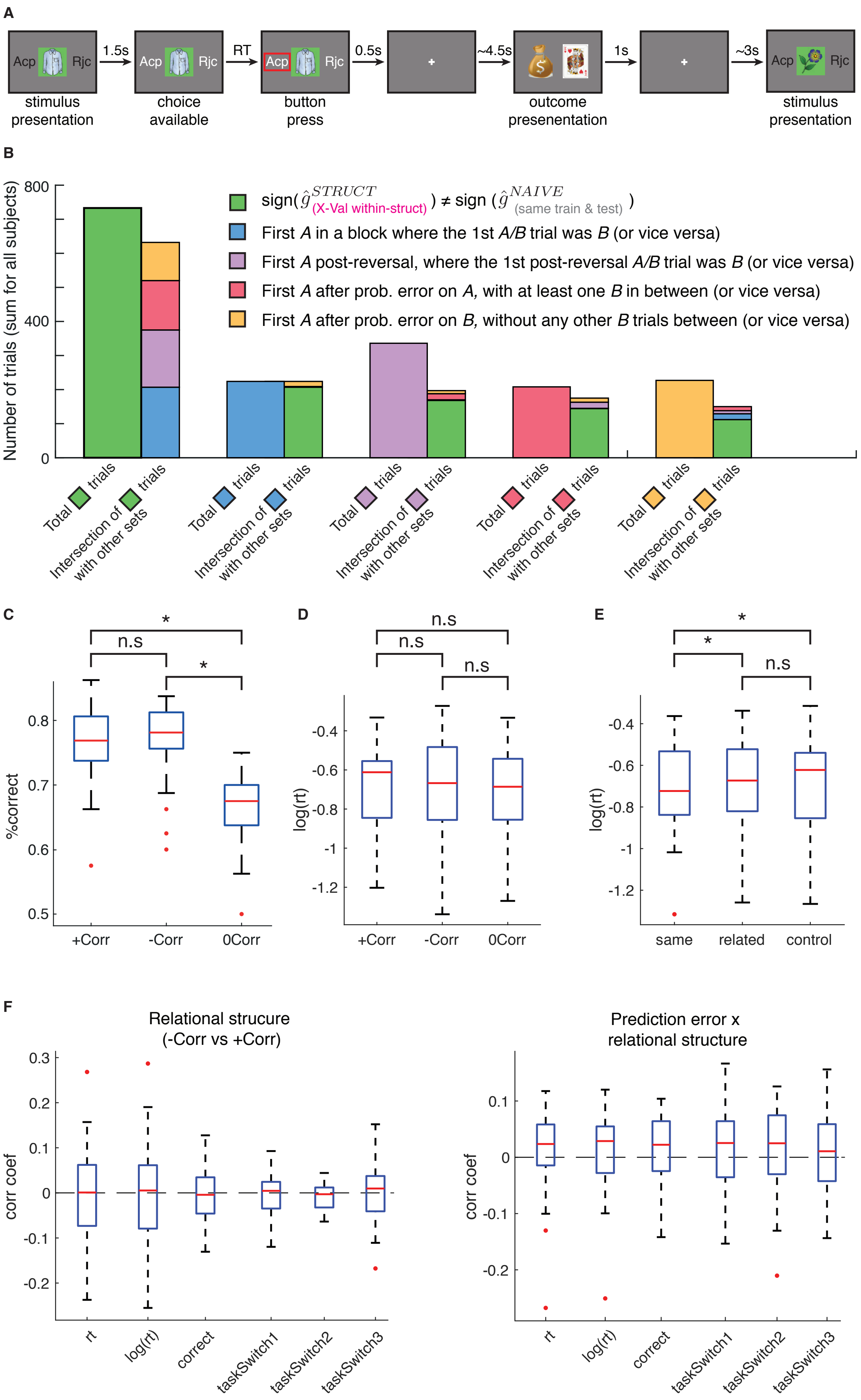
A
Acp
Rjc
1.5s
RT
0.5s
~4.5s
1s
~3s
stimulus presentation
choice available
button press
outcome presenentation
stimulus presentation
B
Number of trials (sum for all subjects)
First A in a block where the 1st A/B trial was B (or vice versa)
First A post-reversal, where the 1st post-reversal A/B trial was B (or vice versa)
First A after prob. error on A, with at least one B in between (or vice versa)
First A after prob. error on B, without any other B trials between (or vice versa)
(X-Val within-struct)
(same train & test)
Total trials
Intersection of trials with other sets
C
D
E
n.s
%correct
log(rt)
+Corr
-Corr
0Corr
same
related
control
F
Relational strucure (-Corr vs +Corr)
Prediction error x relational structure
corr coef
rt
log(rt)
correct
taskSwitch1
taskSwitch2
taskSwitch3

Figure S1. A. Time course of an example trial (related to Figure 1)**.** Acp: Accept; Rjc: reject. RT: reaction time. **B-E: Additional behavioural analyses** (related to Figure 2)**. B.** The trials used in Figure 2B are mostly the first trial in a block where the STRUCT model is useful, or trials following probabilistic errors in outcomes or reversal. We identified 4 sets of trials that constituted the majority of the trials where the within-structure cross-validated STRUCT model (pink elements in Figure 2A; Figure 2B, left) and the NAÏVE model trained and tested on the same data (grey elements in Figure 2A; Figure 2B, right) made different predictions. For each set of the 5 sets (the set of trials used in Figure 2B and the 4 sets identified by their schedule properties), we plotted two bars: the overall number of trials of that set across all participants (left, wider bar), and the number of trials at the intersection of that set with other sets (right, narrower bar). The number of trials at the intersection of any 3 sets was negligible (<14). 86% of the trials used in Figure 2B (green, most left bar) are members of at least one other set. **C.** Subjects performance (measured as % of correct choices, according to the ground truth outcome probability) was better in trials of the related stimuli than trials of the control stimulus (two-tailed paired t-tests: +Corr vs 0Corr t(27)=6.7, P<10^-6; -Corr vs 0Corr paired t(27)=7.11, P<10^-6, -Corr vs +Corr paired t(27)=0.43 P=0.66). **D.** log(reaction times), split by correlation type of trial (-Corr, +Corr or control). Reaction times did not differ between trials of stimuli under different types of correlations (two-tailed paired t-tests: P>0.4 for all comparisons). **E.** Task switching effects. We split all trials to three groups according to the relationship between current and previous trial: “same” (A→A (A trials preceded by an A trial), B→B, C→C), “related” (A→B, B→A) and “control” (A→C, B→C, C→A, C→B), and compared the means of log(reaction times) of these groups across subjects. As expected, subjects were quicker to respond to stimuli preceded by the same stimulus (one-tailed paired t-test on log(reaction times): same vs related: t(27)=2.1, P=0.02, same vs control: t(27)=2.88, P=0.003). However, there was no significant difference between log(reaction times) in trials of stimuli presented after their related stimulus (“related”) compared to trials where stimuli were presented after an unrelated stimulus (“control”), though a weak trend in the expected direction was observed (related vs control: t(27)=0.41, P=0.34). **F. Possible confounds** (related to figures 3 and 4). Correlation coefficients of possible behavioural confounds with the effects of interest. We first constructed six confound regressors: *reaction time*, *log(reaction time)*, *correct* (-1/1 for trials where the subject’s choice was incorrect/correct according to the ground truth outcome probability, respectively), and three task switching regressors where we partitioned the three “task switching groups” (same, related, control – see panel **E**) in different ways, reflecting possible levels of task switching: *taskSwitch1* (“same”: -1, “related”: 0, “control”: 1), *taskSwitch2* (“same” and “related”: -1, “control”: 1) and *taskSwitch3* (“same”: -1, “related” and “control”: 1). Next we constructed regressors reflecting our effects of interest: *relational structure* (-Corr: -1, +Corr: 1, control trials were ignored) and *correctness prediction error x relational structure interaction*. Both regressors showed no significant correlations with any of the confound regressors. In all plots, the red central line is the median, the box edges are the 25th and 75th percentiles, the whiskers extend to the most extreme data points that are not considered outliers, and the outliers are plotted as red circles.

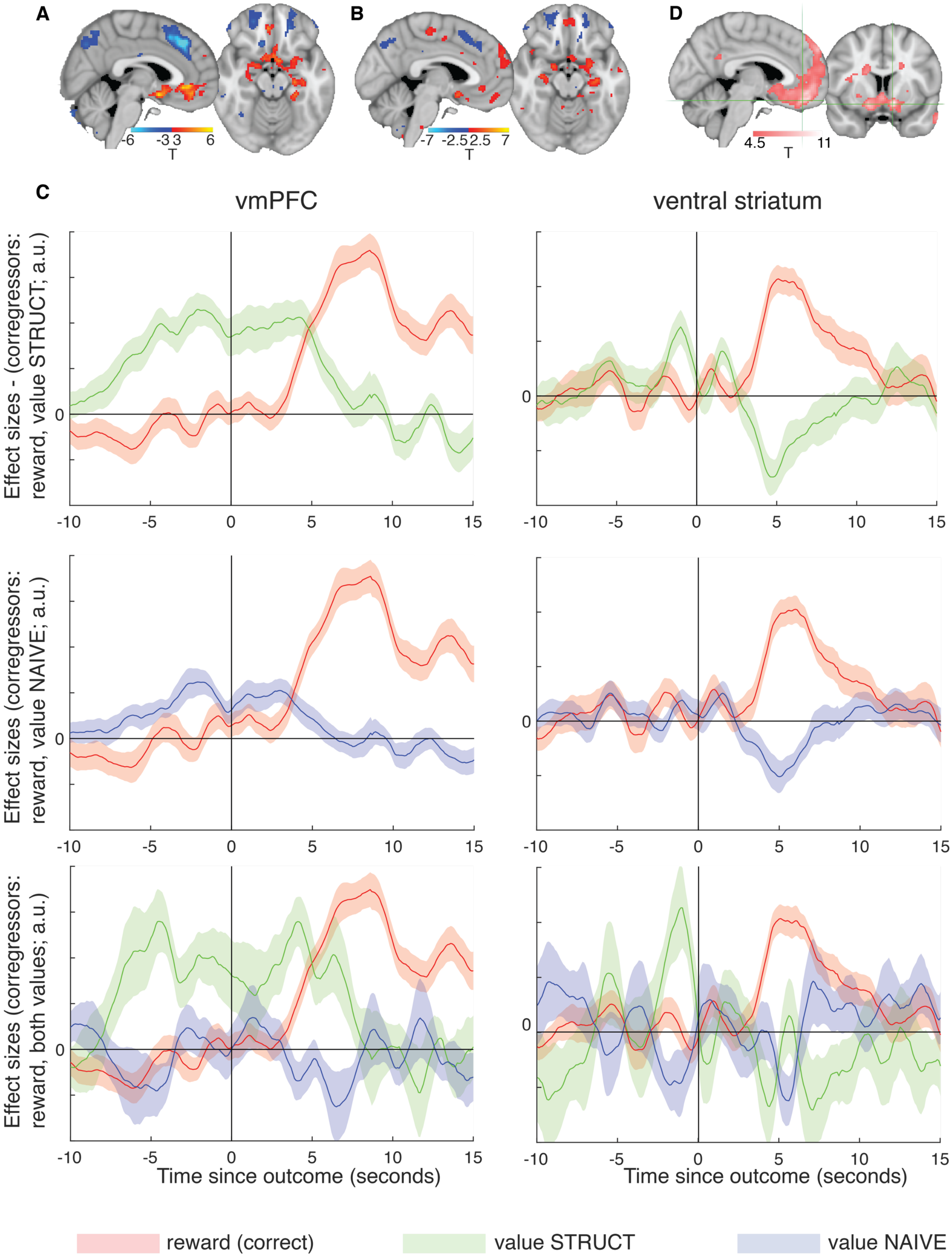

Figure S2. Control analyses for the univariate contrasts (related to "*Additional resources*" section in *STAR methods*, and Figures 2D and 4). **A.** Effect of the contrast [STRUCT chosen action value] > [baseline], locked to stimulus presentation time (same contrast as is Figure 2D), in a GLM that is similar to GLM1 but includes reaction time as a covariate. **B.** Effect of the contrast [STRUCT chosen action value] > [NAÏVE chosen action value], locked to stimulus presentation time, from the same GLM as in Figure 2D (GLM1; note the lower

threshold – no effects survived multiple comparisons correction for this contrast). **C.** Peri-stimulus plots showing the vmPFC (left) and vStr (right) effect time courses of the reward, STRUCT value and NAÏVE value locked to outcome presentation. Time courses are not corrected for the hemodynamic lag. Top: GLM including reward ("correct") and STRUCT chosen action value as co-regressors. Middle: GLM including reward and NAÏVE chosen action value. Bottom: GLM including reward and *both* STRUCT and NAÏVE chosen action values. Prediction error signals (positive reward and negative value effects) can be clearly seen in vStr when the two values do not compete in the same GLM (top and middle right panels). This is also true in vmPFC, once accounting for the sustained value effect from stimulus presentation time (~6.5-8.5 seconds before outcome time) to outcome time (top and middle left panels). However, while the reward effects are still present in a GLM that includes both STRUCT and NAÏVE values, there are no negative value effects due to collinearities between regressors (bottom panels). Data taken from the peaks of the vStr and vmPFC peaks of the STRUCT prediction error effect, used for the prediction error x relational structure interaction effect (Figure 4). **D.** Effect of the contrast [STRUCT correctness prediction error] > [baseline], locked to outcome presentation time (same contrasts as the insets in Figure 4B and 4C), in a GLM that is similar to GLM3 but includes reaction time as a covariate.

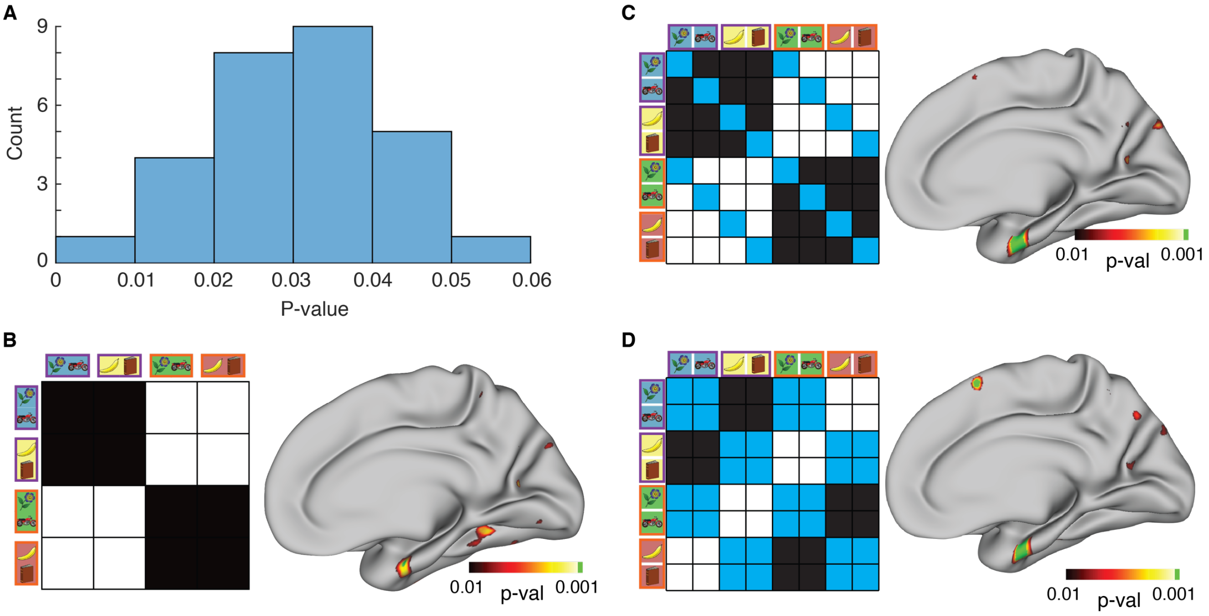

Figure S3. Control contrasts for the EC relational structure effect (related to Figure 3, top)**. A.** Histogram of the leave-one-subject-out cluster-mass FWE-corrected P-values of the EC relational structure effect. **B-D**: Left: model RDMs. Black elements should be similar, white elements should be dissimilar, blue elements are ignored. Right: whole-surface results (right hemisphere). Colour map is the same as in **Figure 3**. B. Relational structure effect from a GLM where the related stimuli (A and B) are collapsed onto the same regressor (GLM2a). **C.** Relational structure effect (GLM2, ignoring all elements of pairs of same-stimulus conditions. **D.** Relational structure effect (GLM2), ignoring all elements of pairs of same stimuli set conditions.

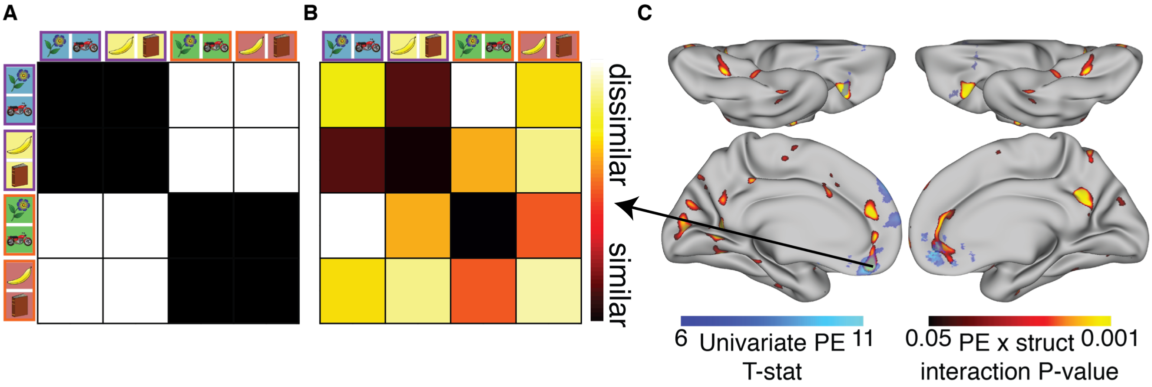

Figure S4. Prediction error x relational structure interaction effect (related to Figure 4)**. A.** Model RDM. Note that each pair of related stimuli correspond to only one condition in the RDM as the trials of each pair are collapsed in GLM3. **B.** Visualisation of the data RDM of the prediction error x relational structure interaction effect at the peak of the vmPFC univariate prediction error effect (MNI [-4,44,-20]). **C.** Visualisation of whole-surface results of the multivariate prediction error x relational structure interaction effect (red-yellow, effects do not survive FWE-correction across a cortical hemisphere - these are exploratory results; note the low threshold), overlaid on the univariate prediction error effect, used to define the ROIs (blue – same as in the insets of Figure 4B and 4C but with a higher threshold). We found bilateral effects in vmPFC (peak P-values: LH: P=0.002 uncorrected, Figure 4A,B; RH: P=0.01 uncorrected, Figure 4C), in close proximity to each hemisphere's univariate peak. The strongest effects in cortex where observed in PCC RH (right hemisphere): P=0.001 uncorrected) and vlPFC (LH: P < 0.001 uncorrected, P=0.002 at the peak of the univariate prediction error vlPFC effect). In all of these regions, the effects were also observed bilaterally, albeit weaker (PCC LH: P = 0.005 uncorrected; vlPFC RH: P=0.005 uncorrected).

Day	Sess	Stimuli Sets per block (each number a single stimuli set - a unique triplet of images. Triplets sampled independently for each subject)	Structure Per block (correlation between outcome probabilities of two stimuli, order counterbalanced across subjects)	Stimuli background color (Blue, Green, Red, Yellow, Counterbalanced across subjects)	Trials per block (same for all subjects)	Feedback (FB, same for all subjects)
1	1	5,5,5,5,5,5,5,5	-,-,-,-,-,-,-,-	None	60	Full
1	2	6,6,6,6,6,6,6,6	-,-,-,-,-,-,-,-	None	60	Full
2	1	7,7,7,7,7,7,7,7	+,+,+,+,+,+,+,+	None	60	Full
2	2	8,8,8,8,8,8,8,8	+,+,+,+,+,+,+,+	None	60	Full
3	1	1,1,1,1,1,1,1,1	-,+,-,+,-,+,-,+	B,G,B,G,B,G,B,G	60	No FB on reject trials in last 15 trials
3	2	2,2,2,2,2,2,2,2	+,-,+,-,+,-,+,-	R,Y,R,Y,R,Y,R,Y	60	No FB on reject trials in last 15 trials
4	1	1,1,2,2,1,1,2,2	-,+,-,+,+,-,+,-	B,G,Y,R,G,B,R,Y	60	No FB on reject trials in last 15 trials
Pre-scan	1	2,2,1,1	-,+,-,+	Y,R,B,G	60	Full
scan	1	1,2,1,2,1,1,2,2	-,+,+,-,-,+,-,+	B,R,G,Y,B,G,Y,R	30	Full

Table S1. Training schedule for an example subject (Related to "*Method detail*" in *STAR Methods*).

2. Model	# parameters	Log likelihood	AIC	BIC
NAÏVE	2 params x<br>2 structures x 28 subjects = 112	-4515.36	9254.72	10077.2
STRUCT	5 params x<br>2 structures x 28 subjects = 280	-2894.44	6348.89	8405.06

Table S2. Formal model comparison between STRUCT and NAÏVE models (related to Figure 2 and to "*Quantification and Statistical Analysis*" section in *STAR methods*). NAIVE parameters: learning rate and inverse temperature. STRUCT parameters: learning rate, inverse temperature and 3 cross-terms.

Peak MNI coords	Brain region(s) of cluster	Peak t-score (df=27)	p-value (FWE-corrected on cluster level)
-32,-16,-20	L anterior hippocampus + amygdala + entorhinal cortex	6.71	P<0.001
-4,10,-12	Posterior mPFC (subcallosal cortex)	6.45	P=0.001
-6,58,36	Dorsal mPFC	4.56	P<0.001
28,-44,64	L Somatosensory cortex	5.56	P=0.006
0,34,-8	vmPFC	5.53	P<0.001
-32,-20,2	Insula/parietal operculum/white matter	5.07	P<0.001
26,4,-22	R hippocampus + amygdala + entorhinal cortex	5	P=0.003
52,-32,20	R parietal operculum	4.88	P<0.001
8,34,42	ACC	6.92	P<0.001
-42,16,6	L Insula	6.68	P<0.001
46,-52,34	R Angular gyrus	6.03	P<0.001
40,44,-2	OFC	5.69	P=0.002

Table S3. Effect of chosen action value of STRUCT model, when competing with NAÏVE model in the same GLM (GLM1, related to Figure 2D). The contrast is [STRUCT chosen action value] > Baseline. All clusters with a FEW-corrected P-value < 0.05 are reported. Note that negative effects (with a negative t-score) are also reported.
