# Supplementary material for: Entorhinal and ventromedial prefrontal cortices abstract and generalize the structure of reinforcement learning problems
Source: Neuron. 2021 Feb 17;109(4):713–723.e7. doi: 10.1016/j.neuron.2020.11.024 (PMC7889496; doi:10.1016/j.neuron.2020.11.024)
Supplement: Document S2. Article plus supplemental information [file mmc2.pdf]

Neuron

Article

Entorhinal and ventromedial prefrontal cortices abstract and generalize the structure of reinforcement learning problems

Highlights

- Entorhinal cortex represents the task structure (rules) of an RL task
- As in physical space, this representation is divorced from sensory signals
- Spatial patterns of prediction error signals depended on task structure
- Such relational representations of task structure might underlie generalisation

Authors

Alon Boaz Baram,
Timothy Howard Muller, Hamed Nili,
Mona Maria Garvert,
Timothy Edward John Behrens

Correspondence

alon.baram@ndcn.ox.ac.uk

In Brief

How do we generalize knowledge between problems that are only loosely related but have a similar structure? Baram et al. take inspiration from the generalization properties of entorhinal cortex in rodent spatial tasks to show that entorhinal and ventromedial prefrontal cortices generalize the structure of reinforcement learning tasks in humans.

Baram et al., 2021, Neuron *109*, 713–723
February 17, 2021 © 2020 The Author(s). Published by Elsevier Inc.
https://doi.org/10.1016/j.neuron.2020.11.024

CellPress

Neuron

CellPress
OPEN ACCESS

Article

Entorhinal and ventromedial prefrontal cortices abstract and generalize the structure of reinforcement learning problems

Alon Boaz Baram,[1,4,*] Timothy Howard Muller,[1] Hamed Nili,[1] Mona Maria Garvert,[1,2] and Timothy Edward John Behrens[1,3]
[1]Wellcome Centre for Integrative Neuroimaging, University of Oxford, John Radcliffe Hospital, Oxford OX3 9DU, UK
[2]Max-Planck-Institute for Human Cognitive and Brain Sciences, Stephanstraße 1a, 04103, Leipzig, Germany
[3]Wellcome Trust Centre for Neuroimaging, University College London, London WC1N 3AR, UK
[4]Lead contact
*Correspondence: alon.baram@ndcn.ox.ac.uk
https://doi.org/10.1016/j.neuron.2020.11.024

SUMMARY

Knowledge of the structure of a problem, such as relationships between stimuli, enables rapid learning and flexible inference. Humans and other animals can abstract this structural knowledge and generalize it to solve new problems. For example, in spatial reasoning, shortest-path inferences are immediate in new environments. Spatial structural transfer is mediated by cells in entorhinal and (in humans) medial prefrontal cortices, which maintain their co-activation structure across different environments and behavioral states. Here, using fMRI, we show that entorhinal and ventromedial prefrontal cortex (vmPFC) representations perform a much broader role in generalizing the structure of problems. We introduce a task-remapping paradigm, where subjects solve multiple reinforcement learning (RL) problems differing in structural or sensory properties. We show that, as with space, entorhinal representations are preserved across different RL problems only if task structure is preserved. In vmPFC and ventral striatum, representations of prediction error also depend on task structure.

INTRODUCTION

Reinforcement learning (RL) theory has given deep insights into the brain's algorithms for learning but has remained relatively mute about the representations that are the foundation for this learning. For example, how might a task, or an element of a task, be represented in the brain? Some recent progress has been made through comparison with spatial navigation, where representations are better understood. It has been suggested that the same representations that map Euclidean space (such as hippocampal place cells [O'Keefe and Dostrovsky, 1971] and entorhinal grid cells [Hafting et al., 2005]) may be extended to a broad range of non-spatial problems. In these cases, instead of representing physical location, they may represent location in an abstract space that captures the regularities of the task at hand (Behrens et al., 2018; Garvert et al., 2017; Gershman and Niv, 2010; Niv, 2019; Schuck et al., 2016; Stachenfeld et al., 2017; Wang et al., 2018; Whittington et al., 2020; Wilson et al., 2014).

One attractive corollary of these ideas is that non-spatial tasks might benefit from the profound representational efficiencies that are known in space. In space, cells in the entorhinal cortex (EC) and adjacent subiculum generalize across different environments and behavioral states. For example, object-vector cells (Høydal et al., 2019) are active when an animal is a specific distance and direction from an object, regardless of the particularities of the object or the environment. Boundary-vector cells (Lever et al., 2009), which fire when an animal is a specific distance and direction from a boundary, show similar invariances to the sensory features of the boundary or the environment. Border cells (Solstad et al., 2008) and boundary cells (Savelli et al., 2008) show similar generalization properties. In "remapping experiments," entorhinal grid cells maintain their cell-cell relationships across environments (Barry et al., 2012; Fyhn et al., 2007; Yoon et al., 2013). This representational structure is even transferred to different behavioral states like sleep (Gardner et al., 2019; Trettel et al., 2019). In new spatial environments, these powerful generalization mechanisms allow immediate transfer of knowledge—it is not necessary to re-learn the associations implied by the structure of 2D space. Instead, it is sufficient to learn what sensory observation is where in the map (Whittington et al., 2020), and inferences (such as shortest paths [Bush et al., 2015; Tolman, 1948]) can be made immediately. These ideas have also been demonstrated causally, by showing that disrupting grid cells' firing impairs path integration (Gil et al., 2018).

The ability to transfer structural knowledge in non-spatial tasks would, similarly, bestow efficiencies. The structure of a problem, learnt in one situation, could be mapped onto a new situation with different sensory observations, and solutions could

 © 2020 The Author(s). Published by Elsevier Inc.
This is an open access article under the CC BY license (http://creativecommons.org/licenses/by/4.0/).

immediately be inferred. Behaviorally, it is clear that both humans and animals profit from such efficiencies. In psychology, this phenomenon is known as "learning-set" (Harlow, 1949)—subjects with prior exposure to the structure of a problem are routinely better at solving new examples.

Might similar mechanisms support both spatial and non-spatial generalization? For this to be true, one prerequisite is that brain regions that contain structural representations in Euclidean spatial tasks should also represent the structure of a non-Euclidean RL task. A second is that, like in Euclidean spaces, these representations should (a) generalize across different sensory exemplars of the same structural problem and (b) differ between two problems of different structures.

To answer these questions, we designed an RL task where we manipulated either the problem structure (by changing the correlation structure of serial bandits) or the sensory stimuli in a 2×2 factorial design. This design mirrors a spatial remapping experiment with two important differences: (1) it is non-spatial task elements that are being remapped, as opposed to locations in 2D space. (2) We include 2 separate structures as well as 2 separate environments (sensory stimuli) in each structure. This design therefore enabled us to test for representations of the task structure, factorized from the representations of the stimuli they were tied to. We hypothesized that the EC will harbor such representations. Though we did not directly compare spatial and non-spatial tasks, this hypothesis was based on the EC's generalization properties in space.

We embedded this factorial design within a variant of a standard RL bandit task. Historically, there has been great success in identifying BOLD correlates of algorithmic RL variables like value and prediction error in such tasks (e.g., Hampton et al., 2006; Ramnani et al., 2004; Wimmer et al., 2012). These findings, however, have been limited to univariate analyses of bulk activity. Our design enabled us to use multivariate techniques to ask new questions about the fine-grained nature of these signals: if learning signals have different consequences in different task structures, they may also conform to the representational predictions in the previous paragraph. Instead of a unitary representation of prediction error, these regions might generalize prediction error representations across different problems with the same task structure but have different representations across problems with different structures.

Using fMRI in humans, we found that the EC contained a representation that differed between different task structures but generalized over different sensory examples of the same structure. Similarly, prediction error signals in regions including vmPFC and ventral striatum maintained different voxelwise patterns for different task structures, again generalizing over different environments with the same structure.

RESULTS

Task

Subjects performed a task where three 1-armed bandits were interleaved pseudo-randomly. Two of the bandits (bandits A and B) had correlated outcome probabilities, while the third (C) was independent. Crucially, we manipulated two features of the task across the different blocks: (1) the sign of the correlation between the A and B bandit probabilities. We refer to this correlation as the relational structure of the stimuli. (2) The stimuli set, with two possible triplets of images. Thus, there were 4 block-types, each with a specific combination of a relational structure and a stimuli set, arranged in a 2×2 factorial design (Figure 1B). The fMRI experiment comprised of 8 blocks of 30 trials each (10 trials per stimulus), divided into 2 independent runs of the 4 block-types, with a pseudo-random block order counterbalanced across subjects. Hence, in total subjects completed 8 blocks, 2 of each block-type.

In each trial, subjects viewed one of the three stimuli and had to indicate their prediction for its associated binary outcome (a "good" or a "bad" outcome, demarked by a King of Hearts or Two of Spades card, respectively) by either accepting or rejecting the stimulus (Figures 1A and S1A). Thus, there was always one correct answer in each trial: subjects should accept a stimulus if they predict the outcome to be the "good" outcome and should reject if they predict the outcome to be the "bad" outcome (Figure 1A). Only in accepted trials, the subject would either win or lose a point, depending on the outcome. Outcome identity was revealed in all trials, including rejection trials, even though the subject's score did not change in these trials (Figure 1A). Predictions of the outcomes could be formed based on the recent history of outcomes. The outcome probabilities switched pseudo-randomly between 0.9 and 0.1 with an average switch probability of 0.15. As the two correlated bandits switched together (Figure 1C), subjects could use their knowledge of the correlation structure to learn from the outcome on one related stimulus about the other.

The outcome probabilities associated with the related stimuli were negatively correlated (−Corr pairs) in half of the blocks (Figure 1C, left two panels), and positively correlated (+Corr pairs) in the other half (Figure 1C, right two panels). In all blocks the third stimulus had an outcome probability which was uncorrelated with the other two stimuli (0Corr pairs). The current block-type was signaled by the background color of all stimuli in the block. Subjects learned the mapping between background color and correlation structure prior to scanning. Consequently, the only learning performed during scanning was of outcome probabilities, not of the relational structure—knowledge of which was available from the first scanning trial.

Behavioral modeling

We modeled the subjects' behavior using an adapted delta-rule, with a crucial addition of "cross-terms" that enable learning from one stimulus to another. Note this is not intended to be a process model of how the brain solves the task. It is intended as a descriptive model that allows us to test whether, and to what extent, subjects are guided by the relational structure of the task. The model separately tracks the probabilities of a "good" outcome associated with the three stimuli, all of which are updated every trial. Following an outcome at trial t where stimulus X was presented, the estimate of the outcome probability associated with X is updated according to the classic delta-rule:

$$\hat{g}^{X}_{t+1} = (1-\alpha)\hat{g}^{X}_{t} + \alpha y_t \qquad \text{(Equation 1)}$$

CellPress
OPEN ACCESS

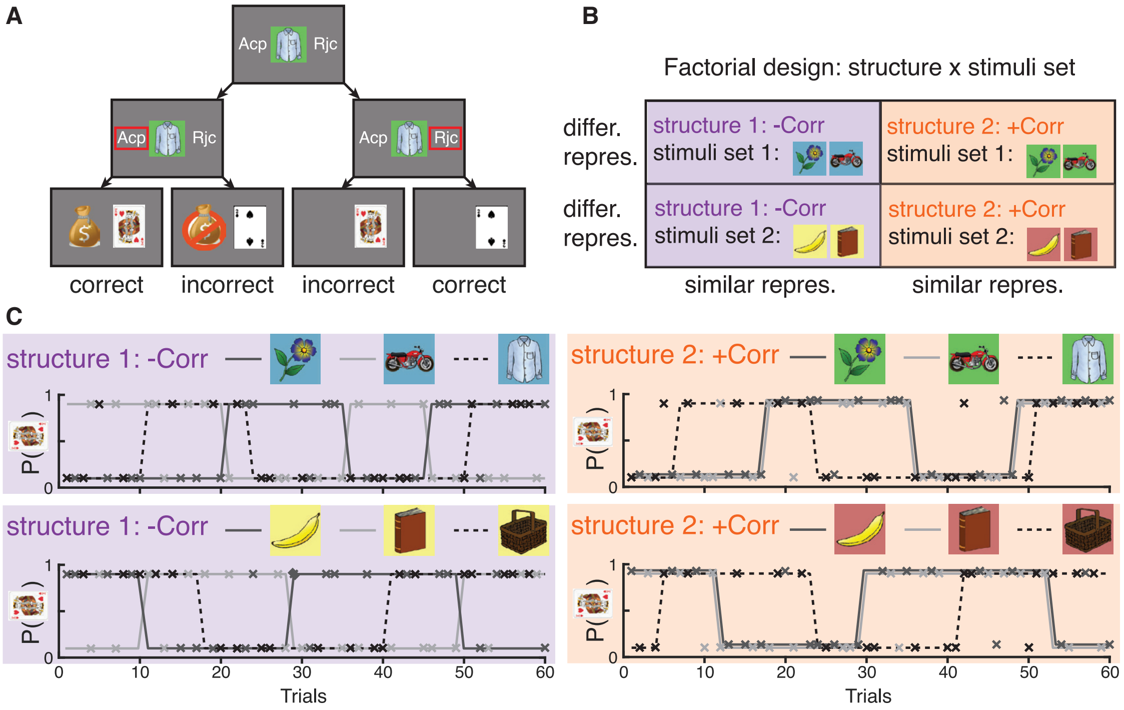

Figure 1. Task design

(A) Possible progressions of a single trial.

(B) Experimental design and neural predictions for structure-encoding brain regions: 2×2 factorial design of stimuli set × relational structure.

(C) Example of the reward schedule for one subject in the four block-types. Solid gray lines and dashed black line are the probabilities of a good outcome for the related stimuli and the control stimulus, respectively. Xs mark the stimuli (color) and actual binary outcomes (y axis: 0.1 and 0.9 are bad and good outcomes, respectively) in each trial. For visualization purposes, the two 30 trials long blocks of each of the four block-types were concatenated. While related stimuli in +Corr blocks (right panels) are associated with exactly the same probability, their corresponding light and dark gray lines are slightly offset for visualization purposes.

where $\hat{g}_t^X$ is the outcome probability estimation for stimulus X before trial t, α is the learning rate, and $y_t \in \{-1, 1\}$ is the binary outcome at trial t.

Crucially, the estimates of the probabilities associated with the two other stimuli are also updated:

$$\hat{g}_{t+1}^Y = (1 - \alpha|H_{XY}|)\hat{g}_t^Y + \alpha H_{XY} y_t \quad \text{(Equation 2)}$$

where H_{XY} is the cross-term between stimuli X and Y, fitted to the subject's behavior in each block. $H_{XY} = 1$ indicates subjects treated X and Y as the same stimulus for learning purposes—for +Corr stimuli pairs, this is the correct correlation structure. Similarly, $H_{XY} = -1$ and $H_{XY} = 0$ indicate correct correlation knowledge for −Corr and 0Corr pairs, respectively. y_t is still the outcome in trial t (a trial where stimulus X was presented).

We henceforth refer to the full model, including the 3 cross-terms, as STRUCT. We also used a structure-naive Rescorla-Wagner model (equivalent to setting the cross-terms in the STRUCT model to 0), which we refer to as NAÏVE.

Subjects' behavior

To determine whether subjects used the relational structure to inform their decisions we employed two approaches: a cross-validation approach and a cross-terms analysis approach. In the cross-validation analysis, for each subject we separately fitted the parameters of the STRUCT and NAÏVE models to data from the 4 block-types, treating trials from each block-type as a separate training set. This resulted in 8 fitted models: 4 STRUCT models and 4 NAÏVE models. We then performed cross-validation by testing how well each of the 8 models predicts subjects' choices in all 4 datasets (Figure 2A). As expected, STRUCT models generalized much better when trained and tested on different datasets of the same relational structure (despite different stimuli, squares highlighted in pink), than when trained on one relational structure and tested on the other (squares highlighted in green; comparison of sum(-log(likelihood)) of pink versus green elements; two-tailed paired t test, $t(27) = 9.54$, $p < 10^{-9}$). Notably, the within-structure cross-validated STRUCT models (squares highlighted in pink) performed better than the NAÏVE models, even when the latter were trained and tested on the same data (squares highlighted in gray; compare pink versus gray elements in Figure 2A: two-tailed paired t test, $t(27) = 4.29$, $p = 0.0002$). To further demonstrate this effect, we plotted the histograms of outcome probability estimations ($\hat{g}$) from the relevant within-structure cross-validated STRUCT model (pink elements in Figure 2A; Figure 2B, left) and the NAÏVE model trained and tested on the same data (gray elements in Figure 2A; Figure 2B, right), split by subjects' choices. When the models made different predictions (see Figure S1B for further characterization of these trials), subjects tended to choose in accordance with the

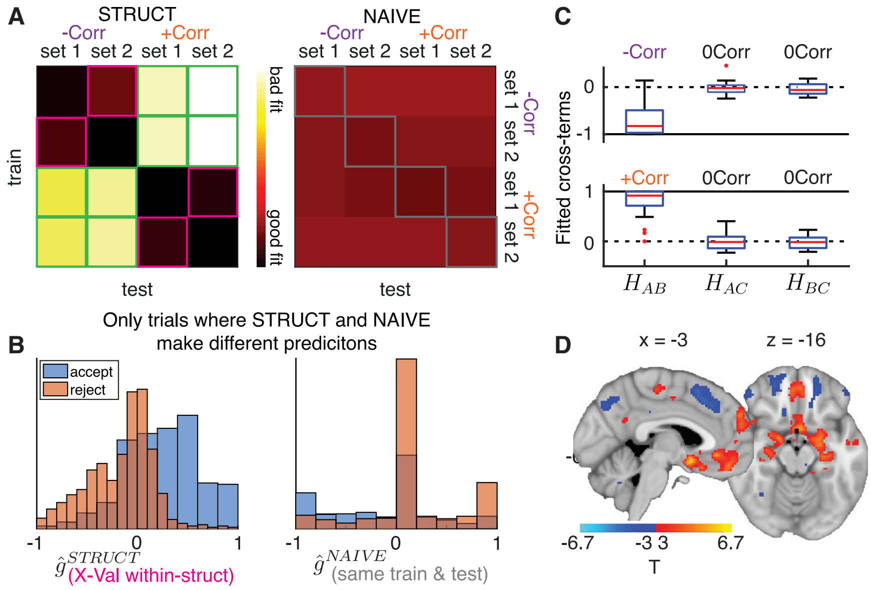

Figure 2. Subjects use the correlation structure correctly

(A) Negative log likelihoods for STRUCT (left) and NAÏVE (right) models (same scale for both matrices). Pink elements: STRUCT models, cross-validated within-structure. Green elements: STRUCT models, cross-validated across structures. Grey elements: NAÏVE models, trained and tested on the same data.

(B) Histograms of the estimated outcome probabilities for trials where subjects accepted (blue) or rejected (orange). Left: STRUCT models trained on data with the same structure but different stimuli set (pink elements in A). Right: NAÏVE models, trained and tested on the same data (gray elements in A). Histograms only include trials where the models make different predictions.

(C) Fitted cross-terms for pairs of stimuli in all −Corr (top) and +Corr (bottom) blocks. Red central line is the median, the box edges are the 25th and 75th percentiles, the whiskers extend to the most extreme datapoints that are not considered outliers, and the outliers are plotted as red circles.

(D) Effect of the chosen action value estimates from STRUCT model, in a GLM where it competes with estimates from NAÏVE model (replication of Hampton et al., [2006]).

within-structure cross-validated STRUCT models (Figure 2B). These results suggest that sensitivity to the correlation structure, here afforded by the cross-terms of STRUCT models, is needed to capture subjects' behavior.

Due to the small number of trials in each block-type, for the analyses in the rest of the manuscript we trained STRUCT and NAÏVE on each subject's concatenated data from all blocks of the same structure. This resulted in 10 (5 parameters × 2 structures) and 4 (2 parameters × 2 structures) fitted parameters per subject for the STRUCT and NAÏVE models, respectively. The fitted cross-terms of the STRUCT model indicated that subjects indeed used the correlation structure correctly (Figure 2C; Corr versus 0Corr (mean across all blocks of $\left|H_{AB}^{-,+}\right|$ versus $(|H_{AC}| + |H_{BC}|)/2$), one-tailed paired t test, t(27) = 13.06, p < 10^−12). The STRUCT model explained subjects' behavior better than the NAÏVE model, even when accounting for the extra free parameters (see a formal model comparison in Table S2). Subjects benefited from the extra information afforded by the correlation structure and performed better in trials of one of the two related stimuli than in the control stimulus trials (Figure S1C). There were no significant differences in reaction times between trials under the three possible correlation types (Figure S1D) and no effect of task switching costs on reaction times between related and unrelated pairs of stimuli (Figure S1E). These results indicate that the subjects who were scanned indeed used the relational structure.

The reward network and the hippocampus use the relational structure to encode the value of the chosen action

Our first aim for the analysis of the fMRI data was to replicate a previous report by Hampton et al. (Hampton et al., 2006), who found evidence of knowledge about the relational structure of the task in known neural signals of RL. We compared how well a model that uses relational structure (STRUCT) explained neural signals relative to one that does not use structure (NAÏVE). In both models, we calculated the value of the chosen action (accept/reject) on each trial of the two related stimuli (A and B). Note that the chosen action value has the same magnitude as the stimulus value tracked by the models but has an opposite sign on rejected trials. The chosen action value estimates were used to construct one regressor per model at the time of stimulus presentation (GLM1). Estimates from both models were pitted against each other in the same GLM, meaning any variance explained by a particular regressor was unique to that regressor, allowing us to compare the neural signals uniquely explained by each model. The contrasts in this section only included the two related stimuli in each block, as the differences between the models for the control stimulus were negligible.

A network of regions including the medial prefrontal cortex (mPFC), the amygdala (AMG), the anterior hippocampus (HPC), and the EC coded positively for the chosen action value from the STRUCT model, while a network including the anterior cingulate cortex (ACC), insula, angular gyrus, and most of the orbitofrontal cortex (OFC) showed negative coding (Figure 2D; Table S3). Similar results were obtained for a GLM that included reaction time as a covariate (Figure S2A) and for the STRUCT > NAÏVE contrast (Figure S2B). This indicates the reward network and HPC use the relational structure to calculate the value of chosen actions. See STAR methods and Figure S2C for a discussion of the equivalent analysis of prediction error signals at outcome time.

Entorhinal representations generalize across tasks with the same structure, but not those with different structures

A representation of the relational structure of the task should be similar (generalize) for stimuli which are part of the same

relational structure, but dissimilar for stimuli under a different relational structure. We asked whether any region on the cortical surface displayed these properties at the times of stimulus presentation, using representational similarity analysis (RSA [Kriegeskorte et al., 2008]) with a searchlight approach.

A searchlight centered on a cortical voxel consisted of the 100 surrounding voxels with the smallest surface-wise geodesic distance from the central voxel. For each searchlight, we obtained 16 patterns of (whitened within-searchlight) regression coefficients of the responses to presentations of each of the two related stimuli (A and B) in each of the 8 blocks (GLM2). In other words, we obtained two patterns, one from each of the repeated runs, for each of our 8 conditions (a particular A or B stimulus under a particular correlation structure). To control for effects of time, we used a "cross-run correlation distance" where only patterns from different runs (i.e., more than 30 min apart) were correlated with each other. That is, to define the distance $d_{i,j}$ between conditions i and j, we first calculated the correlation distance $(1-r)$ between the condition i pattern from run 1 and condition j pattern from run 2, and then calculated the correlation distance between the condition j pattern from run 1 and condition i pattern from run 2. $d_{i,j}$ was defined as the mean of these two distances. Notably, this means that the diagonal in the symmetric representational dissimilarity matrix (RDM) is meaningful and shows the consistency between the two runs of the same condition.

This resulted in an 8-conditions-by-8-conditions symmetric RDM, summarizing the representational geometry in the searchlight (e.g., Figure 3B). The ideal structural representation can be formalized as an 8×8 model RDM, where the distances between conditions are determined by relational structure (Figure 3A). To test whether the data RDM of a given searchlight was consistent with the model RDM, we calculated the contrast between the means of the data RDM's hypothesized "dissimilar" and "similar" elements (white and black elements in Figure 3A, respectively). We verified that this contrast did not correlate with possible behavioral confounds such as reaction time, correctness, or task switching costs (Figure S1F, left). We repeated this procedure for each cortical voxel (searchlight center) of each subject, resulting in a cortical map of contrast values for each subject. To perform group analysis, we projected the maps of all participants to a common cortical surface, resulting in a [number of subjects] long vector of contrast values for each cortical vertex. We then used permutation tests to ask whether this contrast was significantly positive across subjects, resulting in a cortical map of p values that can be corrected for multiple comparisons (e.g., Figure 3D, see STAR methods) or a single p value for an ROI (calculated from the average data RDM across all ROI vertices).

We first tested our *a priori* hypothesis that a representation of the relational structure of the task would be found in EC. Using a searchlight analysis, we found a strong effect in the right EC ($p = 0.005$ small volume corrected for cluster-mass in a right EC anatomical mask (Fischl et al., 2009), $p = 0.01$ after Bonferroni correction for bilateral EC, cluster-forming threshold $p < 0.001$, peak MNI coordinates: [25,−5,−28]). When averaging across the entire anatomical regions defined by the right and left EC masks, there was no effect in left EC ($p = 0.73$) but a highly significant effect in right EC ($p = 0.006$). When averaged across the entire right EC, the structure in the data can be seen by eye and is clearly revealed in dendrograms derived from the distance matrix (Figure 3E). While we did not hypothesize a hemispheric difference, the difference between hemispheres did pass a nominal statistical test ($p = 0.04$). This statistical difference should be treated with caution given the post hoc nature of the test. Although we set out to test an *a priori* ROI, we note that the focal EC cluster revealed by the searchlight analysis was the strongest response in the whole brain (Figures 3B–3D, top, peak MNI coordinates: [25,−5,−28]). The only other notable cluster to survive the cluster-forming threshold of $p < 0.001$ was in the right temporal-parietal junction (TPJ). We verified that the EC effect was not driven by a small number of subjects using a leave-one-out approach (Figure S3A).

The effect was also present in a GLM where we collapsed the two related stimuli in each block onto a single regressor (GLM2a, Figures 3F and S3B). This GLM is somewhat more suited to visualize the relational structure effect, which is blind to stimuli identity: because in this task it is undefined which stimulus is "A" and which is "B," it is not possible to align exact stimuli when comparing across stimuli sets. The effect did not change when we repeated the analysis using model RDMs where same-stimuli or same stimuli set elements were ignored (Figures S3C and S3D). The effect was therefore not driven by background color or low-level plasticity between stimuli that appear in the same block, but rather by a representation of the relational structure between the stimuli in the task.

To verify that our analysis approach was indeed valid, we tested for stimulus visual identity coding using a similar procedure with an appropriate model RDM (Figure 3A, bottom). As expected, we observed bilateral effects in visual areas, peaking in the lateral occipital cortex (LOC, Figures 3B–3D, bottom, $p < 0.001$ FWE corrected for cluster mass across hemisphere, cluster-forming threshold $p < 0.001$, peak MNI coordinates: [44,−74,−4]). The comparison of the EC relational structure effect to a visual object identity effect in LOC can also help to give a sense of the meaningfulness of the RSA measures we are using. The effect of interest (difference between distances of "same" and "different" pairs of conditions, blue bars in Figure 3C) is on average twice as large yet twice as variable for the relational structure contrast at the EC peak (95% confidence intervals = [0.011,0.03]) than the visual identity contrast at the LOC peak (95% confidence intervals = [0.006,0.016]). This shows that the differences in fMRI pattern correlation between conditions in both cases are roughly on the same scale, even though the baseline pattern correlation distances in the peak visual identity LOC searchlight are much smaller than in the peak EC relational structure searchlight (y axis ranges in Figure 3C). The fact that the cluster mass FWE-corrected p value is much lower in the former effect than the latter can be explained by the difference sizes of the regions: the LOC is a much larger region than the EC, and can therefore support larger clusters.

vmPFC and ventral striatum represent the relational structure in learning signals

We hypothesized that vmPFC learning signals of the type typically observed in RL tasks (Hampton et al., 2006; Ramnani et al., 2004; Wimmer et al., 2012) may be sensitive to the

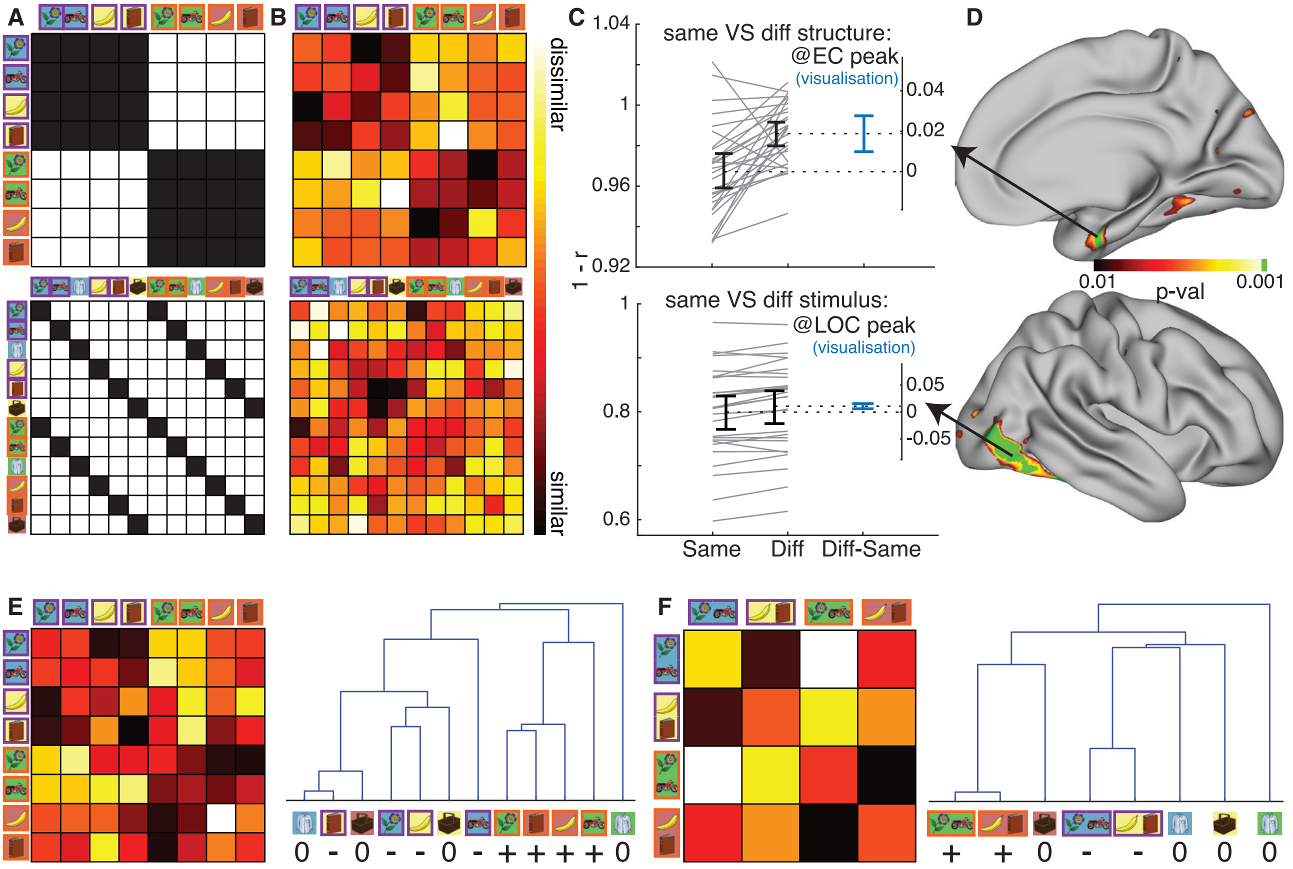

Figure 3. The relational structure of the task is represented in the entorhinal cortex

Top: relational structure effect, peaking in EC. Bottom: stimulus identity effect, peaking in LOC.

(A) Model RDMs. Black elements should be similar, white elements should be dissimilar. Pairs of stimuli with purple and orange rectangles around them are −Corr and +Corr, respectively.

(B) Visualization of the data RDM from peak vertex of the effect, marked with an arrow in (D).

(C) Visualization of the paired mean difference effects between *same* (black RDM elements in A) and *different* (white elements in A) pairs of conditions from the peak vertex of the effects. Both groups are plotted on the left axes as a slope-graph: each paired set of observations for one subject is connected by a line. The paired mean difference is plotted on a floating axis on the right, aligned to the mean of the *same* group. The mean difference is depicted by a dashed line (consequently aligned to the mean of the *diff* group). Error bars indicate the 95% confidence interval obtained by a bootstrap procedure.

(D) Whole surface results, right hemisphere. Clusters surviving FWE correction across the whole surface at a cluster forming threshold of $p < 0.001$ are indicated in green.

(E and F) Average data RDMs (left) across the entire (anatomically defined) right EC, and dendrograms constructed from them (right). (E) Same GLM as in (B–D). (GLM2); (F) A GLM where the two related stimuli in each block were collapsed onto a single regressor (GLM2a). The control stimuli were omitted from the data RDMs for visualization purposes but are included in the dendrograms (labeled “0”).

relational structure. That is, because prediction errors have different implications for learning under the two different relational structures, vmPFC might not only simply encode a signal that monotonically increases with prediction error as previously reported (Hampton et al., 2006; Ramnani et al., 2004; Wimmer et al., 2012). Instead, we hypothesized that the representation of prediction errors across voxels would differ depending on the relational structure.

We first found a strong univariate prediction error signal in a network of regions including vmPFC (inset Figures 4B–4C; Figure S2D left) and the ventral striatum (vStr, inset Figure 4D; Figure S2D right), in line with previous findings (Hampton et al., 2006; Ramnani et al., 2004; Wimmer et al., 2012). Note that the prediction error used here is the “correctness” prediction error, defined as the magnitude of the prediction error from the STRUCT model, and a sign that depends on the congruence between the subject’s choice and the outcome: positive when the outcome matches the subject’s choice (accept–“good” outcome; reject–“bad” outcome), and negative when choice and outcome are incongruent (accept–“bad” outcome; reject–“good” outcome). This prediction error is exactly the same as a classic reward prediction error, under the assumption that avoiding punishment (correct reject trial) and gaining a reward (correct accept trial) are equivalent.

We next asked whether the multivoxel pattern of this prediction error signal depends on the relational structure on a

CellPress
OPEN ACCESS

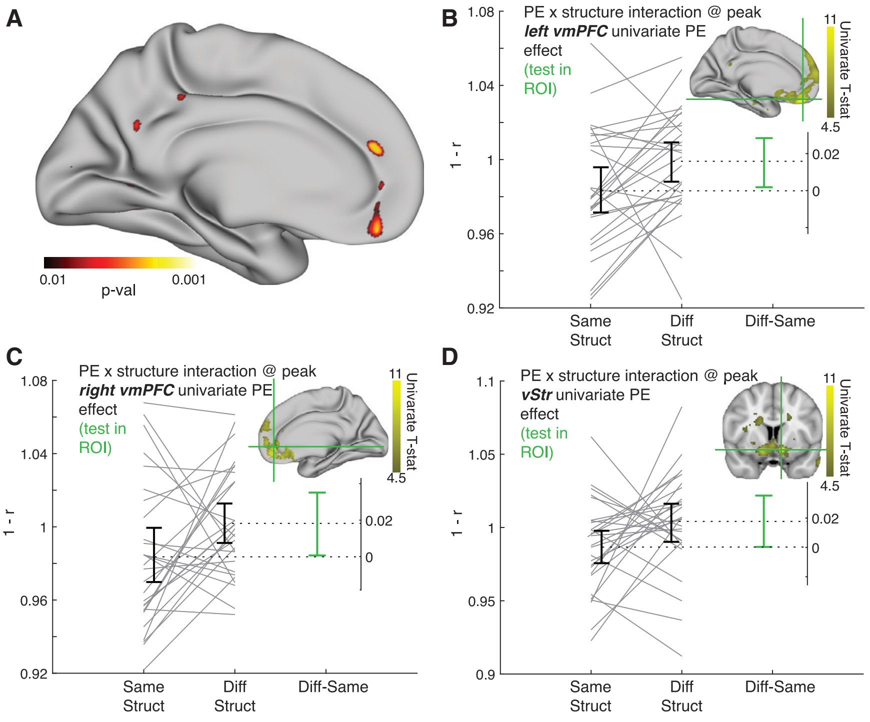

Figure 4. Prediction error signals in vmPFC and ventral striatum depend on the current relational structure of the task

(A) Visualization of whole-surface results of the multivariate prediction error × relational structure interaction effect, medial left hemisphere.

(B) Interaction effect at the left hemisphere vmPFC peak of the univariate prediction error effect (MNI: [−4,44,−20]).

(C) Interaction effect at the right hemisphere vmPFC peak of the univariate prediction error effect (MNI: [8,44,−11]).

(D) Interaction effect at the ventral striatum peak univariate prediction error effect (MNI: [−10,8,−12]). Brain images in the insets of (B), (C), and (D) show the univariate prediction error effect (projected on the surface in B and C). Legend for (B), (C), and (D) is the same as in Figure 3C.

fine-grained scale, in a multivariate analysis. We conducted a searchlight RSA analysis similar to the one from the previous section, with two notable differences: (1) the patterns used as inputs to the RSA were not the average responses to the stimuli, but instead the regression coefficients of the prediction errors on the two related stimuli (A and B) in each block. This means the patterns entering this analysis are the local spatial variations in the representation of prediction errors. (2) These analyses only have a single measure per block as opposed to two (separate stimuli) in Figures 3B–3E. This is similar to GLM2a (Figure 3F), but with prediction error parametric regressors at outcome time rather than onset regressors at stimulus presentation time. The resulting RDMs are therefore 4×4, not 8×8 (Figures S4A and S4B).

Because we are testing the multivariate differences on the (orthogonal) univariate prediction error effect, we could use the peaks of the univariate effect to constrain our regions of interest. The top three univariate peaks were in bilateral vmPFC (left hemisphere (LH) peak MNI [−2,48,−18], t(27) = 9.3, inset of Figure 4B and Figure S4C left; right hemisphere (RH) peak MNI [8,44,−11], t(27) = 9.36, inset of Figure 4C and Figure S4C right) and vStr (peak MNI [−10,8,−12], t(27) = 10.24, inset of Figure 4D). In searchlights centered on these peaks, the multivariate prediction error × structure interaction effect was significant (LH vmPFC: p = 0.014, Figures 4A, 4B, S4B, and S4C left; RH vmPFC: p = 0.02, Figures 4C and S4C right; vStr: p = 0.034, Figure 4D). See Figure S4C for exploratory results of this effect in a bilateral reward-related network including posterior cingulate cortex (PCC) and ventrolateral PFC (Rushworth et al., 2011). As with the relational structure effect, we verified its interaction with prediction errors did not correlate with any behavioral confound (Figure S1F, right). In addition, our results held when we ran the GLM using an HRF with a delay that was fitted to the peak of the univariate prediction error signal (delay of 8.5 s, instead of the default delay of 6 s, see STAR methods and Figure S2C for details).

It is important to note that here we do not test for the *existence* of structural information in the prediction error signals, as we do for value signals in Figure 2D (see additional resources for a discussion of the equivalent analysis for prediction errors). Rather, the results in this section indicate that there is *spatial variation* in the prediction error signals in vmPFC and vStr (and perhaps vlPFC and PCC) that depends on the current relational structure of the task. The critical difference between the two relational structures in our experiment is not how the prediction error should be computed, but rather how it should be used to inform future behavior—how should "credit" for the error be assigned (Gershman et al., 2015; Gershman and Niv, 2010; Jocham et al., 2016; Rudebeck et al., 2017; Walton et al., 2010; Wilson et al., 2014). One intriguing possibility is therefore that different representations of prediction errors allow different credit assignment for the same prediction errors in the two relational structures. Factorizing these computations from the sensory particularities of the task allows them to be rapidly "remapped" to new stimuli for rapid learning.

DISCUSSION

Understanding how the brain represents abstract task state-spaces remains a major challenge. Our data join growing evidence suggesting state-space representations rely on the hippocampal formation (Garvert et al., 2017; Miller et al., 2017; Stachenfeld et al., 2017; Vikbladh et al., 2019; Wang et al., 2020; Zhou et al., 2019b) and interconnected regions in the ventral PFC (Schuck et al., 2016; Vaidya et al., 2020; Wang et al., 2020; Wilson et al., 2014; Zhou et al., 2019a, 2019b). This is particularly interesting in light of the historical role of these regions in generalization and relational reasoning (Barron et al., 2013; Bowman and Zeithamova, 2018; Cohen and Eichenbaum,

1993; Morrissey et al., 2017; Preston and Eichenbaum, 2013; Zeithamova and Preston, 2010), which are essential for efficient task representations. Here, we show that these regions generalize the relational structure of a non-Euclidian RL task. EC representations of stimuli generalized over tasks with the same structure, but not over tasks with a different structure. The same was true for vmPFC (and vStr) representations of prediction error. These results suggest a common framework for the representation and generalization of task structures in a wide variety of domains.

Our experiment can be viewed as a set of non-spatial "remapping experiments." The vertical arms in Figure 1B (same task structure, different stimuli) are analogous to the classic spatial sensory remapping experiments in rodents (Barry et al., 2012; Fyhn et al., 2007), where an animal is moved between different sensory examples of the same task structure (namely free foraging in a Euclidean 2D space). In these experiments, entorhinal grid cells maintain (generalize) their covariance structure across environments (i.e., they do not remap, in contrast to hippocampal place cells [Bostock et al., 1991]). Such generalization was also observed in Macaque OFC neurons, across different sensory examples of an economic decision-making task (Xie and Padoa-Schioppa, 2016). The horizontal arms in Figure 1B (same stimuli, different task structure) can be viewed as "task remapping" experiments. In the spatial case of such experiments, where animals are required to perform different tasks in the same sensory environment, recent evidence suggests the grid code changes (remaps) across tasks (Boccara et al., 2019; Butler et al., 2019). Our EC effect (Figure 3) mirrors these results in a non-spatial RL task in humans. Future work might address whether the exact same neuronal population underlies generalization in both spatial and non-spatial tasks.

We believe the comparison to spatial EC cells that generalize over sensory particularities (e.g., object-vector, boundary-vector, boundary, and border cells), and in particular grid cells as their most studied example, is essential to understand our results: first, grid cells abstract and generalize the relational structure of 2D environments. This is demonstrated in remapping tasks (Barry et al., 2012; Fyhn et al., 2007; Whittington et al., 2020), which inspired the design of our task. Second, an important property of the relational structure that they generalize is the correlation structure of observations, that is shared between all 2D environments, even when the observations themselves differ. Theoretical work suggests the hexagonal grid representation (Banino et al., 2018; Dordek et al., 2016; Mathis et al., 2015; Stachenfeld et al., 2017), and other EC spatial representation such as boundary, boundary-vector, and object-vector cells (Whittington et al., 2020), are obtained from low dimensional projections of correlation structures that are subject to the constraints of 2D. Here, we similarly manipulate the correlation structure of observations, only these observations are rewards in an abstract RL task rather than locations in a spatial task. Third, grid cells reside predominantly in EC, but also in human mPFC (Jacobs et al., 2013) (and other areas)—the areas where we find our effects. However (and crucially), we do not claim that hexagonal grid-like patterns (Bao et al., 2019; Constantinescu et al., 2016; Doeller et al., 2010) underlie our effects, nor that it is possible to measure these here. A hexagonal representation, while being the most efficient representation for generalizing the relational properties of 2D Euclidean space (Banino et al., 2018; Dordek et al., 2016; Mathis et al., 2015; Stachenfeld et al., 2017), would not be useful for generalization in our task. Rather, we test—in a non-Euclidean RL state-space—for the underlying computational functions (reviewed in Behrens et al. [Behrens et al., 2018; Dordek et al., 2016; Stachenfeld et al., 2017; Whittington et al., 2020]) that lead to the hexagonal pattern in 2D space.

A unified framework for the representation of task structure might also afford a new way to interpret standard RL neural signals like prediction error. While the dependence of prediction error signals on prior, unobservable information has been reported previously (Nakahara et al., 2004), here we report a novel relational aspect to this context dependency. Prediction error representations that were used in different ways (opposite update signs of the related stimulus for −/+Corr blocks) differed anatomically on a fine-grained level. Our vmPFC prediction error × structure interaction effect (Figure 4) brings together observations about vmPFC function from several seemingly disparate fields, including RL (Rushworth et al., 2011). Patients with vmPFC lesions show a selective valuation deficit when value comparison should be based on attribute configuration, i.e., when relationships between object elements are important for the valuation (Pelletier and Fellows, 2019). In the memory literature, vmPFC has been strongly implicated in the representation of schemas—abstract structures of previous knowledge, which bear many parallels to the relational structures discussed here (Baldassano et al., 2018; Gilboa and Marlatte, 2017). vmPFC is particularly important when new information is assimilated into an existing schema (Sommer, 2017; Tse et al., 2011), analogous to a prediction error update of the internal model of the task within the current structure. Finally, some of the strongest effects of spatial and nonspatial grid-like coding in fMRI were recorded in the medial PFC (Constantinescu et al., 2016; Doeller et al., 2010; Jacobs et al., 2013). Notably PCC, where we found the strongest prediction error × structure interaction effect in our exploratory analysis (Figure S4C), also exhibits grid-like coding (Constantinescu et al., 2016; Doeller et al., 2010) and has also been strongly implicated in the representations of schemas (Baldassano et al., 2018; Sommer, 2017).

Though our manuscript focuses on novel data regarding structural representations of task elements (Figure 3) and learning signals (Figure 4), we also report a key replication (Figure 2D). This is notable, as the effect that is replicated is subtle—the unique contribution of model-based (over model-free) value in a relational RL task. However, in the original paper (Hampton et al., 2006), only a mPFC effect was reported in a GLM that included chosen action values from (the equivalents of) *both* STRUCT and NAÏVE models. The other brain regions (amygdala, anterior hippocampus, EC) where we found a positive STRUCT chosen action value effect in such a GLM were reported by Hampton et al. in a weaker analysis—in a GLM including *only* estimates from (the equivalent of) the STRUCT model. We also report the negative effects of the contrast from the more stringent GLM (in angular gyrus, ACC and OFC, where negative correlates of value are often observed). It is plausible that the effects we report

here also existed in Hampton et al.'s data and were not reported due to the focus of their manuscript on mPFC and the tendency at the time not to report negative effects.

Learning can be dramatically improved by a useful representation of the world you are learning about. Here, we show that the brain can "recycle" (generalize) these representations, enabling fast and flexible inferences. We believe the comparison of generalizable representations in our abstract RL task to their parallels in spatial cognition is a useful one and can suggest a path for a more precise understanding of the nature of these representations.

STAR★METHODS

Detailed methods are provided in the online version of this paper and include the following:

- KEY RESOURCES TABLE
- RESOURCE AVAILABILITY
    - Lead contact
    - Materials availability
    - Data and code availability
- EXPERIMENTAL MODEL AND SUBJECT DETAILS
    - Participants
- METHOD DETAILS
    - Training and task
- QUANTIFICATION AND STATISTICAL ANALYSIS
    - Behavior modeling
    - fMRI data acquisition
    - Pre-processing
    - Univariate analyses
    - RSA analyses
    - Multiple comparisons correction
    - Leave-one-out analysis - EC effect
- ADDITIONAL RESOURCES
    - The univariate "correctness" prediction error signal
    - Characteristics of the HRF in vmPFC

SUPPLEMENTAL INFORMATION

Supplemental Information can be found online at https://doi.org/10.1016/j.neuron.2020.11.024.

ACKNOWLEDGMENTS

We thank Jacob Bakermans, Anna Shpektor, Philipp Schwartenbeck, Avital Hahamy, and Shirley Mark for useful comments on earlier versions of the manuscript. We thank the following funding sources: Clarendon scholarship to A.B.B; MRC scholarship to T.H.M.; Max Planck Society support to M.M.G; Wellcome Senior Research Fellowship (104765/Z/14/Z), Wellcome Principal Research Fellowship (219525/Z/19/Z), and JS McDonnell Foundation award (JSMF220020372) to T.E.J.B.; the Wellcome Centre for Integrative Neuroimaging is supported by core funding from the Wellcome Trust (203139/Z/16/Z).

AUTHOR CONTRIBUTIONS

A.B.B., T.H.M., and T.E.J.B. designed the study; A.B.B. acquired the data; A.B.B. and T.E.J.B. analyzed the data, with input from T.H.M., M.M.G., and H.N; A.B.B. and T.E.J.B. wrote and edited the manuscript.

DECLARATION OF INTERESTS

The authors declare no conflicting interests.

Received: August 3, 2020
Revised: October 9, 2020
Accepted: November 19, 2020
Published: December 22, 2020

REFERENCES

Baldassano, C., Hasson, U., and Norman, K.A. (2018). Representation of Real-World Event Schemas during Narrative Perception. J. Neurosci. *38*, 9689–9699.

Banino, A., Barry, C., Uria, B., Blundell, C., Lillicrap, T., Mirowski, P., Pritzel, A., Chadwick, M.J., Degris, T., Modayil, J., et al. (2018). Vector-based navigation using grid-like representations in artificial agents. Nature *557*, 429–433.

Bao, X., Gjorgieva, E., Shanahan, L.K., Howard, J.D., Kahnt, T., and Gottfried, J.A. (2019). Grid-like Neural Representations Support Olfactory Navigation of a Two-Dimensional Odor Space. Neuron *102*, 1066–1075.e5.

Barron, H.C., Dolan, R.J., and Behrens, T.E.J. (2013). Online evaluation of novel choices by simultaneous representation of multiple memories. Nat. Neurosci. *16*, 1492–1498.

Barry, C., Ginzberg, L.L., O'Keefe, J., and Burgess, N. (2012). Grid cell firing patterns signal environmental novelty by expansion. Proc. Natl. Acad. Sci. USA *109*, 17687–17692.

Behrens, T.E.J., Muller, T.H., Whittington, J.C.R., Mark, S., Baram, A.B., Stachenfeld, K.L., and Kurth-Nelson, Z. (2018). What Is a Cognitive Map? (Organizing Knowledge for Flexible Behavior. Neuron).

Boccara, C.N., Nardin, M., Stella, F., O'Neill, J., and Csicsvari, J. (2019). The entorhinal cognitive map is attracted to goals. Science *363*, 1443–1447.

Bostock, E., Muller, R.U., and Kubie, J.L. (1991). Experience-dependent modifications of hippocampal place cell firing. Hippocampus *1*, 193–205.

Bowman, C.R., and Zeithamova, D. (2018). Abstract memory representations in the ventromedial prefrontal cortex and hippocampus support concept generalization. J. Neurosci. *38*, 2605–2614.

Bush, D., Barry, C., Manson, D., and Burgess, N. (2015). Using Grid Cells for Navigation. Neuron *87*, 507–520.

Butler, W.N., Hardcastle, K., and Giocomo, L.M. (2019). Remembered reward locations restructure entorhinal spatial maps. Science *363*, 1447–1452.

Cohen, N.J., and Eichenbaum, H. (1993). Memory, Amnesia, and the Hippocampal System (Cambridge: MIT Press).

Constantinescu, A.O., O'Reilly, J.X., and Behrens, T.E.J. (2016). Organizing conceptual knowledge in humans with a gridlike code. Science *352*, 1464–1468.

Dale, A.M., Fischl, B., and Sereno, M.I. (1999). Cortical surface-based analysis. I. Segmentation and surface reconstruction. Neuroimage *9*, 179–194.

Delgado, M.R., Nystrom, L.E., Fissell, C., Noll, D.C., and Fiez, J.A. (2000). Tracking the hemodynamic responses to reward and punishment in the striatum. J. Neurophysiol. *84*, 3072–3077.

Desikan, R.S., Ségonne, F., Fischl, B., Quinn, B.T., Dickerson, B.C., Blacker, D., Buckner, R.L., Dale, A.M., Maguire, R.P., Hyman, B.T., et al. (2006). An automated labeling system for subdividing the human cerebral cortex on MRI scans into gyral based regions of interest. Neuroimage *31*, 968–980.

Doeller, C.F., Barry, C., and Burgess, N. (2010). Evidence for grid cells in a human memory network. Nature *463*, 657–661.

Dordek, Y., Soudry, D., Meir, R., and Derdikman, D. (2016). Extracting grid cell characteristics from place cell inputs using non-negative principal component analysis. eLife *5*, e10094.

Fischl, B. (2012). FreeSurfer. Neuroimage *62*, 774–781.

Fischl, B., Sereno, M.I., and Dale, A.M. (1999). Cortical surface-based analysis. II: Inflation, flattening, and a surface-based coordinate system. Neuroimage *9*, 195–207.

Fischl, B., Liu, A., and Dale, A.M. (2001). Automated manifold surgery: constructing geometrically accurate and topologically correct models of the human cerebral cortex. IEEE Trans. Med. Imaging *20*, 70–80.

Fischl, B., Salat, D.H., Busa, E., Albert, M., Dieterich, M., Haselgrove, C., van der Kouwe, A., Killiany, R., Kennedy, D., Klaveness, S., et al. (2002). Whole brain segmentation: automated labeling of neuroanatomical structures in the human brain. Neuron *33*, 341–355.

Fischl, B., Stevens, A.A., Rajendran, N., Yeo, B.T.T., Greve, D.N., Van Leemput, K., Polimeni, J.R., Kakunoori, S., Buckner, R.L., Pacheco, J., et al. (2009). Predicting the location of entorhinal cortex from MRI. Neuroimage *47*, 8–17.

Fyhn, M., Hafting, T., Treves, A., Moser, M.-B., and Moser, E.I. (2007). Hippocampal remapping and grid realignment in entorhinal cortex. Nature *446*, 190–194.

Gardner, R.J., Lu, L., Wernle, T., Moser, M.-B., and Moser, E.I. (2019). Correlation structure of grid cells is preserved during sleep. Nat. Neurosci. *22*, 598–608.

Garvert, M.M., Dolan, R.J., and Behrens, T.E.J. (2017). A map of abstract relational knowledge in the human hippocampal-entorhinal cortex. eLife *6*, 1–20.

Gershman, S.J., and Niv, Y. (2010). Learning latent structure: carving nature at its joints. Curr. Opin. Neurobiol. *20*, 251–256.

Gershman, S.J., Norman, K.A., and Niv, Y. (2015). Discovering latent causes in reinforcement learning. Curr. Opin. Behav. Sci. *5*, 43–50.

Gil, M., Ancau, M., Schlesiger, M.I., Neitz, A., Allen, K., De Marco, R.J., and Monyer, H. (2018). Impaired path integration in mice with disrupted grid cell firing. Nat. Neurosci. *21*, 81–91.

Gilboa, A., and Marlatte, H. (2017). Neurobiology of Schemas and Schema-Mediated Memory. Trends Cogn. Sci. *21*, 618–631.

Hafting, T., Fyhn, M., Molden, S., Moser, M.-B., and Moser, E.I. (2005). Microstructure of a spatial map in the entorhinal cortex. Nature *436*, 801–806.

Hampton, A.N., Bossaerts, P., and O'Doherty, J.P. (2006). The role of the ventromedial prefrontal cortex in abstract state-based inference during decision making in humans. J. Neurosci. *26*, 8360–8367.

Harlow, H.F. (1949). The formation of learning sets. Psychol. Rev. *56*, 51–65.

Ho, J., Tumkaya, T., Aryal, S., Choi, H., and Claridge-Chang, A. (2019). Moving beyond P values: data analysis with estimation graphics. Nat. Methods *16*, 565–566.

Høydal, Ø.A., Skytøen, E.R., Andersson, S.O., Moser, M.B., and Moser, E.I. (2019). Object-vector coding in the medial entorhinal cortex. Nature *568*, 400–404.

Jacobs, J., Weidemann, C.T., Miller, J.F., Solway, A., Burke, J.F., Wei, X.-X.X., Suthana, N., Sperling, M.R., Sharan, A.D., Fried, I., and Kahana, M.J. (2013). Direct recordings of grid-like neuronal activity in human spatial navigation. Nat. Neurosci. *16*, 1188–1190.

Jenkinson, M., and Smith, S. (2001). A global optimisation method for robust affine registration of brain images. Med. Image Anal. *5*, 143–156.

Jenkinson, M., Bannister, P., Brady, M., and Smith, S. (2002). Improved optimization for the robust and accurate linear registration and motion correction of brain images. Neuroimage *17*, 825–841.

Jenkinson, M., Beckmann, C.F., Behrens, T.E.J., Woolrich, M.W., and Smith, S.M. (2012). FSL. Neuroimage *62*, 782–790.

Jocham, G., Brodersen, K.H., Constantinescu, A.O., Kahn, M.C., Ianni, A.M., Walton, M.E., Rushworth, M.F.S., and Behrens, T.E.J. (2016). Reward-Guided Learning with and without Causal Attribution. Neuron *90*, 177–190.

Kleiner, M., Brainard, D., Pelli, D., Ingling, A., Murray, R., and Broussard, C. (2007). What's new in psychtoolbox-3. Perception *36*, 1–16.

Kriegeskorte, N., Goebel, R., and Bandettini, P. (2006). Information-based functional brain mapping. Proc. Natl. Acad. Sci. USA *103*, 3863–3868.

Kriegeskorte, N., Mur, M., and Bandettini, P. (2008). Representational similarity analysis - connecting the branches of systems neuroscience. Front. Syst. Neurosci. *2*, 4.

Lever, C., Burton, S., Jeewajee, A., O'Keefe, J., and Burgess, N. (2009). Boundary vector cells in the subiculum of the hippocampal formation. J. Neurosci. *29*, 9771–9777.

Mathis, A., Stemmler, M.B., and Herz, A.V.M. (2015). Probable nature of higher-dimensional symmetries underlying mammalian grid-cell activity patterns. eLife *4*, 1–29.

Miller, K.J., Botvinick, M.M., and Brody, C.D. (2017). Dorsal hippocampus contributes to model-based planning. Nat. Neurosci. *20*, 1269–1276.

Morrissey, M.D., Insel, N., and Takehara-Nishiuchi, K. (2017). Generalizable knowledge outweighs incidental details in prefrontal ensemble code over time. eLife *6*, 1–20.

Nakahara, H., Itoh, H., Kawagoe, R., Takikawa, Y., and Hikosaka, O. (2004). Dopamine neurons can represent context-dependent prediction error. Neuron *41*, 269–280.

Nichols, T.E., and Holmes, A.P. (2002). Nonparametric permutation tests for functional neuroimaging: a primer with examples. Hum. Brain Mapp. *15*, 1–25.

Nili, H., Wingfield, C., Walther, A., Su, L., Marslen-Wilson, W., and Kriegeskorte, N. (2014). A toolbox for representational similarity analysis. PLoS Comput. Biol. *10*, e1003553.

Niv, Y. (2019). Learning task-state representations. Nat. Neurosci. *22*, 1544–1553.

O'Doherty, J.P., Dayan, P., Friston, K., Critchley, H., and Dolan, R.J. (2003). Temporal difference models and reward-related learning in the human brain. Neuron *38*, 329–337.

O'Keefe, J., and Dostrovsky, J. (1971). The hippocampus as a spatial map. Preliminary evidence from unit activity in the freely-moving rat. Brain Res. *34*, 171–175.

Pelletier, G., and Fellows, L.K. (2019). A critical role for human ventromedial frontal lobe in value comparison of complex objects based on attribute configuration. J. Neurosci. *39*, 4124–4132.

Penny, W.D., Friston, K.J., Ashburner, J.T., Kiebel, S.J., and Nichols, T.E. (2007). Statistical parametric mapping: the analysis of functional brain images (Elsevier).

Preston, A.R., and Eichenbaum, H. (2013). Interplay of hippocampus and prefrontal cortex in memory. Curr. Biol. *23*, R764–R773.

Ramnani, N., Elliott, R., Athwal, B.S., and Passingham, R.E. (2004). Prediction error for free monetary reward in the human prefrontal cortex. Neuroimage *23*, 777–786.

Rescorla, R.A., and Wagner, A.R. (1972). A theory of Pavlovian conditioning: Variations in the effectiveness of reinforcement and nonreinforcement. In Classical Conditioning II: Current Research and Theory, A.H. Black and W.F. Prokasy, eds. (Appleton Century Crofts), pp. 64–99.

Reuter, M., Rosas, H.D., and Fischl, B. (2010). Highly accurate inverse consistent registration: a robust approach. Neuroimage *53*, 1181–1196.

Rudebeck, P.H., Saunders, R.C., Lundgren, D.A., and Murray, E.A. (2017). Specialized Representations of Value in the Orbital and Ventrolateral Prefrontal Cortex: Desirability versus Availability of Outcomes. Neuron *95*, 1208–1220.e5.

Rushworth, M.F.S., Noonan, M.P., Boorman, E.D., Walton, M.E., and Behrens, T.E.J. (2011). Frontal cortex and reward-guided learning and decision-making. Neuron *70*, 1054–1069.

Savelli, F., Yoganarasimha, D., and Knierim, J.J. (2008). Influence of boundary removal on the spatial representations of the medial entorhinal cortex. Hippocampus *18*, 1270–1282.

Schuck, N.W., Cai, M.B., Wilson, R.C., and Niv, Y. (2016). Human Orbitofrontal Cortex Represents a Cognitive Map of State Space. Neuron *91*, 1402–1412.

Ségonne, F., Dale, A.M., Busa, E., Glessner, M., Salat, D., Hahn, H.K., and Fischl, B. (2004). A hybrid approach to the skull stripping problem in MRI. Neuroimage *22*, 1060–1075.

Smith, S.M. (2002). Fast robust automated brain extraction. Hum. Brain Mapp. *17*, 143–155.

CellPress
OPEN ACCESS

Solstad, T., Boccara, C.N., Kropff, E., Moser, M.-B., and Moser, E.I. (2008). Representation of geometric borders in the entorhinal cortex. Science *322*, 1865–1868.

Sommer, T. (2017). The Emergence of Knowledge and How it Supports the Memory for Novel Related Information. Cereb. Cortex *27*, 1906–1921.

Stachenfeld, K.L., Botvinick, M.M., and Gershman, S.J. (2017). The hippocampus as a predictive map. Nat. Neurosci. *20*, 1643–1653.

Tolman, E.C. (1948). Cognitive maps in rats and men. Psychol. Rev. *55*, 189–208.

Trettel, S.G., Trimper, J.B., Hwaun, E., Fiete, I.R., and Colgin, L.L. (2019). Grid cell co-activity patterns during sleep reflect spatial overlap of grid fields during active behaviors. Nat. Neurosci. *22*, 609–617.

Tse, D., Takeuchi, T., Kakeyama, M., Kajii, Y., Okuno, H., Tohyama, C., Bito, H., and Morris, R.G.M. (2011). Schema-dependent gene activation and memory encoding in neocortex. Science *333*, 891–895.

Vaidya, A.R., Jones, H.M., Castillo, J., and Badre, D. (2020). Neural representation of abstract task structure during generalization. bioRxiv. Published online July 21, 2020. https://doi.org/10.1101/2020.07.21.213009.

Vikbladh, O.M., Meager, M.R., King, J., Blackmon, K., Devinsky, O., Shohamy, D., Burgess, N., and Daw, N.D. (2019). Hippocampal Contributions to Model-Based Planning and Spatial Memory. Neuron *102*, 683–693.e4.

Walton, M.E., Behrens, T.E.J., Buckley, M.J., Rudebeck, P.H., and Rushworth, M.F.S. (2010). Separable learning systems in the macaque brain and the role of orbitofrontal cortex in contingent learning. Neuron *65*, 927–939.

Wang, J.X., Kurth-Nelson, Z., Kumaran, D., Tirumala, D., Soyer, H., Leibo, J.Z., Hassabis, D., and Botvinick, M. (2018). Prefrontal cortex as a meta-reinforcement learning system. Nat. Neurosci. *21*, 860–868.

Wang, F., Schoenbaum, G., and Kahnt, T. (2020). Interactions between human orbitofrontal cortex and hippocampus support model-based inference. PLoS Biol. *18*, e3000578.

Whittington, J.C.R., Muller, T.H., Mark, S., Chen, G., Barry, C., Burgess, N., and Behrens, T.E.J. (2020). The Tolman-Eichenbaum Machine: Unifying space and relational memory through generalization in the hippocampal formation. Cell *183*, 1249–1263.e23.

Wilson, R.C., Takahashi, Y.K., Schoenbaum, G., and Niv, Y. (2014). Orbitofrontal cortex as a cognitive map of task space. Neuron *81*, 267–279.

Wimmer, G.E., Daw, N.D., and Shohamy, D. (2012). Generalization of value in reinforcement learning by humans. Eur. J. Neurosci. *35*, 1092–1104.

Winkler, A.M., Ridgway, G.R., Webster, M.A., Smith, S.M., and Nichols, T.E. (2014). Permutation inference for the general linear model. Neuroimage *92*, 381–397.

Xie, J., and Padoa-Schioppa, C. (2016). Neuronal remapping and circuit persistence in economic decisions. Nat. Neurosci. *19*, 855–861.

Yoon, K., Buice, M.A., Barry, C., Hayman, R., Burgess, N., and Fiete, I.R. (2013). Specific evidence of low-dimensional continuous attractor dynamics in grid cells. Nat. Neurosci. *16*, 1077–1084.

Zeithamova, D., and Preston, A.R. (2010). Flexible memories: differential roles for medial temporal lobe and prefrontal cortex in cross-episode binding. J. Neurosci. *30*, 14676–14684.

Zhou, J., Gardner, M.P.H., Stalnaker, T.A., Ramus, S.J., Wikenheiser, A.M., Niv, Y., and Schoenbaum, G. (2019a). Rat Orbitofrontal Ensemble Activity Contains Multiplexed but Dissociable Representations of Value and Task Structure in an Odor Sequence Task. Curr. Biol. *29*, 897–907.

Zhou, J., Montesinos-Cartagena, M., Wikenheiser, A.M., Gardner, M.P.H., Niv, Y., and Schoenbaum, G. (2019b). Complementary Task Structure Representations in Hippocampus and Orbitofrontal Cortex during an Odor Sequence Task. Curr. Biol. *29*, 3402–3409.e3.

STAR★METHODS

KEY RESOURCES TABLE

REAGENT or RESOURCE	SOURCE	IDENTIFIER
Software and algorithms		
MATLAB v2016a	Mathworks	https://www.mathworks.com
FSL v6.0	Jenkinson et al., 2012	https://fsl.fmrib.ox.ac.uk/fsl/fslwiki
SPM12	Penny et al., 2007	https://www.fil.ion.ucl.ac.uk/spm/software/
Psychtoolbox3	Kleiner et al., 2007	http://psychtoolbox.org/
RSA toolbox	Nili et al., 2014	https://git.fmrib.ox.ac.uk/hnili/rsa
Freesurfer	Fischl, 2012	https://surfer.nmr.mgh.harvard.edu/
DABEST	Ho et al., 2019	https://acclab.github.io/DABEST-python-docs/index.html

RESOURCE AVAILABILITY

Lead contact

Further information and requests for resources and reagents should be directed to and will be fulfilled by the Lead Contact, Alon B. Baram (alon.baram@ndcn.ox.ac.uk).

Materials availability

This study neither used any reagent nor generated new materials.

Data and code availability

All code is available at https://github.com/alonbaram2/relationalStructure. Unthresholded statistical maps can be obtained at https://neurovault.org/collections/7150.

EXPERIMENTAL MODEL AND SUBJECT DETAILS

Participants

We trained 49 volunteers over 4 days on an online version of the task. 17 subjects did not proceed to be scanned as they either did not comply with task demands (e.g., failed to complete training on time) or did not reach a behavioral criterion for knowledge of the outcome probabilities correlation structure (a difference of more than 0.3 between the fitted cross-term of the related stimuli and the mean of the fitted cross-terms of the unrelated stimuli, see below). 32 volunteers (aged 21-32 years, mean age 23.4, 18 females) with normal or corrected-to-normal vision and no history of neurological or psychiatric disorders participated in the fMRI experiment. 4 subjects were excluded from the analyses: 3 due to technical difficulties during the scanning, and one due to excessive motion. Hence, all analyses presented are based on data from 28 subjects. All subjects gave written informed consent and the study was approved by the University of Oxford ethics committee (reference: R51215/RE001).

METHOD DETAILS

Training and task

Subjects trained online for 4 days prior to the scan day, and were scanned on day 5. In each training session, subjects performed a task where three 1-armed bandits were interleaved pseudo-randomly. The bandits were cued by three different visual stimuli, randomly sampled without replacement for each session from a bank of 35 images. Two of the bandits (bandits A & B) had correlated outcome probabilities, while the third (C) was independent. There were two possible correlation structures for the outcome probabilities of bandits A & B: positive correlation (+Corr blocks) or negative correlation (-Corr blocks). Each online training session comprised of 8 blocks with 60 trials each (3 stimuli X 20 trials per stimulus).

In each trial, subjects viewed one of the three stimuli and had to indicate their prediction for its associated binary outcome (a “good” or a “bad” outcome, demarked by a King of Hearts or Two of Spades card, respectively) by either accepting or rejecting the stimulus. Thus, there was always one correct answer in each trial: subjects should accept a stimulus if they predict the outcome to be the “good” outcome, and should reject if they predict the outcome to be the “bad” outcome (Figure 1A). Only in accepted trials, the subject would either win or lose a point, depending on the outcome (i.e., accepting incorrectly resulted in a loss of a point).

Outcome identity was revealed in all trials, including rejection trails (except on training days 3 and 4, see below), even though the subject's score did not change in these trials (Figure 1A). Predictions of the outcomes could be formed based on the recent history of outcomes. The outcome probabilities switched pseudo-randomly between 0.9 and 0.1 with an average switch probability of 0.05 in each trial of the training sessions. As the two correlated bandits switched together, subjects could use their knowledge of the correlation structure to learn from the outcome on stimulus A about stimulus B and vice versa (Figure 1C). Subjects were informed and reminded at the beginning of each training block that two of the bandits had correlated outcome probabilities, and that this correlation might be positive or negative. However, subjects had to infer which two bandits were correlated and what the sign of the correlation was.

The training schedule for an example subject is shown in Table S1. On day 1 subjects completed two sessions with a different triplet of stimuli used as the ABC cues in each session. In both sessions, two of the stimuli had a particular correlation structure, counterbalanced across subjects. That is, half of the subjects performed two sessions of 8 -Corr blocks each, while the other half performed two sessions of 8 +Corr blocks. Day 2 was identical to day 1, except that the two stimuli sets used were novel and the correlation structure was the one that the subject did not experienced on day 1. On day 3 subjects again completed 2 sessions with a novel stimuli set per session, where the correlation structure between two of the stimuli alternated between blocks. The correlation structure was indexed by the background color of stimuli (e.g., Figures 1B and 1C), and subjects were informed that the combination of stimuli set, background color and correlation structure in day 3 will be the same in days 4 and 5, including in the scanning task. Thus, subjects could already learn the background color-correlation structure mapping prior to entering the scanner. On day 4 subjects completed one session with all the 4 possible block-types (2 stimuli sets × 2 correlation structures, e.g., Figures 1B and 1C). To reduce available information and facilitate subjects' need to use the extra information afforded by the correlation structure, no counterfactual feedback was given on rejection trials in any of the last 15 trials of a block in training days 3 and 4.

Prior to scanning on day 5, subjects completed a pre-scanning reminder session with all 4 block-types, again with the same stimuli set - background color - correlation structure combinations as in days 3 and 4. In both the pre-scanning session and during scanning, full outcome feedback was given, including in all rejection trials. During scanning, subjects completed 8 blocks of 30 trials each (10 trials per stimulus), with a break after 4 blocks for structural and field-maps scans. The two groups (runs) of 4 blocks included one of each of the 4 experimental block-types in a pseudo-random order, counterbalanced across subjects. Outcome probabilities switched faster in the scanner than during training due to the shorter blocks, with a switch probability of 0.15.

Before the first trial of each block, all three stimuli and the background color of that block were presented. A trial was on average 11.5 s long, progressing in the following order (Figure S1A): 1) A stimulus would appear in the middle of the screen, together with the available choices, e.g., left for accept (corresponding to an index finger button press) and right to reject (middle finger button press). The left/right mapping to choices was counterbalanced across subjects but stable within-subject. 2) After 1.5 s, the frame of the stimulus and the accept/reject text turned white, indicating that choice can now be made. Subjects who indicated their choice prior to the appearance of the white frame lost half a point. 3) Subjects indicated their choice by pressing either the index or middle finger buttons. Next, a red rectangle appeared around the chosen option for 0.5 s. 4) A white fixation cross appeared for a variable period, drawn from an exponential distribution with a mean of 4.5 s and truncated between 3.5-5.5 s. The purpose of this long delay between choice and outcome was to enable the independent analysis of both periods, due to the sluggish nature of the hemodynamic response function. 5) The outcome of the trial appeared for 1 s in the middle of the screen - either the "good" outcome (King of Hearts card) or the "bad" outcome (Two of Spades card). If the subject has accepted the trial earlier, they will either win or lose a point: if they accepted correctly (outcome was "good"), a "sack of gold" image would appear in the left side of the screen to indicate that a point was gained; if they accepted incorrectly (outcome was "bad"), a "no sack of gold" image would appear to indicate that a point was lost. If the subject rejected the trial, no points would be won or lost (and hence no "sack of gold" or "no sack of gold" image would appear), but the outcome card image would still appear (Figure 1A). Hence subjects received full counterfactual feedback. Note that subjects should reject trials if they predict the outcome to be the "bad" outcome, as they will lose a point for incorrectly accepting a trial. 6) A white fixation cross appeared for a variable inter trial interval, drawn from an exponential distribution with a mean of 3 s and truncated between 2.5-4 s.

QUANTIFICATION AND STATISTICAL ANALYSIS

Behavior modeling

We modeled the behavior of the subjects using an adapted delta-rule model (Rescorla and Wagner, 1972). The original delta-rule model estimates the outcome probabilities for a given stimulus using the following equation:

$\widehat{g}_{t+1}^{S} = \widehat{g}_{t}^{S} + \alpha\varepsilon_{t}^{S}; \varepsilon_{t}^{S} = y_t - \widehat{g}_{t}^{S}$, where $S \in \{A,B,C\}$; $\{A,B,C\}$ are the three stimuli presented in a block, $\widehat{g}_{t}^{S}$ is the value estimation of stimulus S before trial t (which can be thought of as the "good" outcome probability estimation for stimulus S before trial t, mapped to the [-1,1] interval instead of [0,1]), α is the learning rate, ε is the outcome prediction error, and y_t is the outcome at trial t: $y_t = 1$ for the "good" outcome and $y_t = -1$ for the "bad" outcome. Note that the model estimates the stimulus value, based on the outcome identity (which card was obtained - not the reward actually obtained by the subject), and is agnostic to the choice the subject made. The stimulus value should be distinguished from the "chosen action value" used in GLM1 (see below), which has the same magnitude as

the stimulus value, but has an opposite sign on rejection trials: in a trial where the subject's hypothetical estimate of the stimulus value is very low (close to -1), they will be confident in rejecting the trial, making the value of the chosen "reject" action high (close to $+1$). Similarly, ε should not to be confused with the "correctness" prediction error used in GLM3 and Figure 4, which has the same magnitude as ε but with a sign determined by the congruence between the subject's choice and the outcome. The stimulus value estimation can then be used by a "selector model" to make a choice in the next trial, by using a simple sigmoidal function: $P(\textit{choice on stimulus S on trial } t+1 = \textit{accept}) = \left(1+e^{-\beta(\widehat{g}_t^S)}\right)^{-1}$, where β is the inverse temperature, controlling the randomness of the choice. In this model, α and β are free parameters fit to subjects' behavior.

However, this model does not use any knowledge about the correlations between the outcome probabilities of different stimuli. To allow for this, we added three free parameters to the model, which we refer to as cross-terms. These parameters determine how information on one stimulus affects the outcome estimate on another stimulus.

Following an outcome on stimulus A, we update the outcome estimates of all three stimuli:

$$\widehat{g}_{t+1}^A = (1-\alpha)\widehat{g}_t^A + \alpha y_t$$

$$\widehat{g}_{t+1}^B = (1-\alpha|H_{AB}|)\widehat{g}_t^B + \alpha H_{AB} y_t$$

$$\widehat{g}_{t+1}^C = (1-\alpha|H_{AC}|)\widehat{g}_t^C + \alpha H_{AC} y_t$$

Where $-1 \le H_{XY} \le 1$ is the cross-term for stimuli X and Y. Note that the first equation (update of the estimate for stimulus A following an outcome on stimulus A) is identical to the update in the original delta-rule model. $H_{XY} = 1$ means stimuli X and Y are treated as the same: the outcome estimates for both stimuli will be updated in exactly the same way following feedback on one of them. $H_{XY} = -1$ means stimuli X and Y are treated as having opposite (anti-correlated) outcome probabilities. $H_{XY} = 0$ means the two outcome probabilities are treated as uncorrelated: the outcome estimates for stimulus X will not change following feedback on stimulus Y, and vice versa. Analogous updates occur when feedback is given on stimuli B or C.

To establish the robustness of the STRUCT model in explaining subjects' choices, we first performed a cross-validation analysis. We fitted both STRUCT (5 free parameters per training data: learning rate, inverse temperature and 3 cross-terms) and NAÏVE (2 free parameters per training data: learning rate and inverse temperature) models to training data from the four block-types (2 structures × 2 stimuli sets) separately, concatenating data from the pre-scanning (1 block of 42 trials per block type) and scanning (2 blocks of 30 trials per block type) sessions. Hence, we fitted 4 STRUCT models and 4 NAÏVE models. We then tested the trained models on data of subjects' choices from either the same (diagonals in Figure 2A) or different (off-diagonals in Figure 2A) block-type, resulting in 4×4 matrices of the negative log likelihood of the test data given the fitted model (Figure 2A). The outcome estimates for all stimuli were reset to 0 at the beginning of each block. We fit the parameters by maximizing the negative log likelihood of the data with respect to the parameters using the MATLAB function *fmincon.* The learning rate was constrained to be between 0 and 1, the cross-terms between -1 and 1, and the inverse temperature between 0 and 8.

To further demonstrate the robustness of the STRUCT model, we isolated trials where the NAÏVE model, when trained and tested on the same data (gray elements in Figure 2A, right), predicted different choices than the cross-validated STRUCT model (trained and tested on different data but from the same relational structure, pink elements in Figure 2A, left). We plotted histograms of the models' outcome probability estimation, separately for accept/reject choices of subjects.

For the rest of the paper, we used STRUCT and NAÏVE models fitted on data pooled across all blocks of the same structure (collapsing over stimuli sets). That is, we collapsed data from the subject's scanning session (30 trials per block × 4 blocks per structure) and the pre-scanning session (42 trials per block × 2 blocks per structure), and fitted the parameters separately for +Corr and -Corr blocks. This resulted in a total of 202 trials for each of the structures (+/−Corr). The final STRUCT and NAIVE models had 10 (5 parameters × 2 structures) and 4 (2 parameters × 2 structures) free parameters per subject, respectively. We confirmed that the STRUCT model provided a better fit to the data even when accounting for the extra degrees of freedom through a formal model comparison (Table S2). Finally, we tested whether subjects indeed used the relational structure correctly by performing a one-tailed paired t test across subjects between the STRUCT model AB cross-term and the mean of AC and BC cross-terms (Figure 2C).

We note that we do not suggest that this model reflects how the brain solves the task. The model is only used as a way to analyze the behavioral and neural data. Specifically, the model allows us to 1) Establish that the relational structure influenced subjects' behavior. 2) Compute a proxy to value and prediction error signals, to use in the fMRI analysis.

fMRI data acquisition

Data were acquired on a 3T Siemens Prisma scanner, using a 32-channel head coil. Functional scans were collected using a T2*-weighted echo-planar imaging (EPI) sequence with a multi-band acceleration factor of 3, within-plane acceleration factor (iPAT) of 2, TR = 1.235 s, TE = 20 ms, flip angle = 65 degrees, voxel resolution of 2×2×2 mm and a tilt of 30 degrees relative to axial axis. A field map with dual echo-time images (TE1 = 4.92ms, TE2 = 7.38ms, whole-brain coverage, voxel size 3×3×3 mm) was acquired

to correct for geometric distortions. Structural scans were acquired using a T1-weighted MP-RAGE sequence with 1×1×1 mm voxels.

Pre-processing

Pre-processing was performed using tools from the fMRI Expert Analysis Tool (FEAT), part of FMRIB's Software Library (FSL (Jenkinson et al., 2012)). Data for each of the 8 blocks were pre-processed separately. Each block was aligned to the first, pre-saturated image using the motion-correction tool MCFLIRT (Jenkinson et al., 2002). Brain extraction was preformed using automated brain extraction tool BET (Smith, 2002). All data were high-pass temporally filtered with a cut-off of 100 s. Registration of EPI images to high-resolution structural images and to standard (MNI) space was performed using FMRIB's Linear and Non-Linear Registration Tool (FLIRT and FNIRT (Jenkinson et al., 2002; Jenkinson and Smith, 2001)), respectively. The registration transformations were then used to move each blocks' EPI data to the native structural space, downsampled to 2×2×2 resolution. No spatial smoothing was performed during pre-processing (see below for different smoothing protocols for univariate and multivariate analyses).

Univariate analyses

Due to incompatibility of FSL with the MATLAB RSA toolbox (Nili et al., 2014) used in subsequent analyses, we estimated all first-level GLMs and univariate group-level analyses using SPM12 (Wellcome Trust Centre for Neuroimaging, https://www.fil.ion.ucl.ac.uk/spm).

For univariate analyses, contrasts of parameter estimates were smoothed with a kernel of 5mm FWHM before performing group level statistics.

In the following descriptions, [A, B, C] refer to the three stimuli presented in a particular block, where the outcome probabilities associated with A & B were correlated (either positively or negatively, depending on the block). Regressors in all GLMs were modeled as delta functions (stick), convolved with the default SPM HRF (hemodynamic delay of 6 s).

To test whether known RL signals in the reward network were consistent with a model that used the relational structure, we constructed a GLM where chosen action value estimates from both STRUCT and NAÏVE models were pitted against each other (without being orthogonolised, Figures 2D and S2). GLM1 included the following regressors per each block: two main effect regressors of all related stimuli trials ([AB]): one modeling the times of stimulus presentation and modeling outcome times. Two parametric regressors were locked to stimulus presentation times of [AB]: the value of the chosen option from the STRUCT and the NAÏVE models. In addition, the GLM included several other regressors: C trials at stimulus presentation; C trials at outcome; 2 regressors modeling button presses across all stimuli: one modeling all "accept" trials and one modeling all "reject" trials; 6 motion parameters as nuisance regressors; bias term modeling the mean activity in each block. Figure 1E shows the results of the contrast [STRUCT chosen option value] > [baseline]. Figure S2B shows the results of the contrast [STRUCT chosen option value] > [NAIVE chosen option value]. Figure S2A shows the results of the [STRUCT chosen option value] > [baseline] contrast in a separate GLM where we added [AB trials reaction times] as a parametric regressor locked to the time of stimulus presentation.

See below for a description of the univariate STRUCT model prediction error analysis, the results of which are shown in the insets of Figures 4B and 4C.

RSA analyses

In this section, we first outline the steps common to all RSA (Kriegeskorte et al., 2008) analyses, and then describe details which were specific to each analysis. All RSA analyses were conducted as follows: 1) A searchlight (Kriegeskorte et al., 2006) was constructed around each cortical voxel, including the 100 cortical voxels with the smallest surface-wise geodesic distance from the central voxel. This was performed using adapted scripts from the RSA toolbox (Nili et al., 2014 original surface-based searchlight scripts written by Joern Diedrichsen & Naveed Ejaz, code available at https://github.com/rsagroup/rsatoolbox) . The searchlight definition depends on cortical reconstruction and alignment performed via Freesurfer's *recon-all* command (Dale et al., 1999; Desikan et al., 2006; Fischl et al., 1999, 2002, 2001; Reuter et al., 2010; Ségonne et al., 2004). Defining cortical searchlights on the 2D surface (rather than in 3D) is useful as it is sensitive to the subject-specific anatomy of the cortical folding. 2) For each searchlight, first-level univariate GLMs on unsmoothed data were conducted for all voxels in the searchlight using the RSA toolbox, based on SPM12. Regression coefficients were then spatially pre-whitened within the searchlight using the RSA toolbox. 3) A distance metric was defined to summarize the representational geometry between conditions. The metrics used were either the cross-run correlation distance or a within-block correlation distance (See below). This resulted in data RDMs of size [number of conditions] × [number of conditions]. 4) A hypothesis about the representational geometry was formalised as a contrast between the mean of RDM elements that should "dissimilar" to each other and the mean of RDM elements that should be "similar" to each other. This contrast was calculated in each searchlight, resulting in a single contrast map per subject. 5) The contrast maps of the different subjects were aligned on the common cortical surface (consisting of vertices, rather than voxels) using Freesurfer-based scripts adapted from the RSA toolbox. 6) Group-level statistical significance of the contrast was performed either in an a-priori ROI or in a whole-cortex manner. For the former (Figures 3E and 3F), for each subject we first averaged the RDMs within the ROI and then performed a one-tail t test on the contrasts values across participants). For the latter (all other RSA results) we performed statistical inference (equivalent to a one-tailed t test) and family-wise error (FWE) correction using permutation tests (Nichols and Holmes, 2002) using PALM (Winkler et al., 2014). In this procedure, the contrast values from each subject were randomly multiplied by either 1 or −1, following the null hypothesis that the contrasts are

symmetric around 0. The test's statistic was then defined as the cross-subject average of contrast values. This was repeated 10000 times, creating a null distribution of the means. The true value of this mean was then compared to this null distribution. The resulting (uncorrected) p value map is displayed in Figures 3D, 4A, S3, and S4C as a heatmap, at a threshold of $p < 0.01$. The paired mean difference across subjects between the two groups of RDM elements at particular vertices of interest is visualized in the Gardner-Altman estimation plots in Figures 3C, 4B, and 4C. The figures were generated using an adaptation of the openly available MATLAB package DABEST (Ho et al., 2019). We make an important distinction between estimation plots containing data from peaks of FWE-surviving clusters, which are subject to selection bias (and are shown for visualization purposes only; Figure 3C), and estimation plots with data from unbiased ROIs, which are not subject to selection bias (Figures 4B and 4C). In these ROIs an uncorrected statistical test can be performed.

To search for a representation of the relational structure between stimuli in the task (Figures 3 and S3), we conducted a GLM (GLM2) which included the following regressors per each block: 3 main effect regressors ([A],[B],[C]) modeling the times of stimulus presentation; 3 main effect regressors ([A], [B], [C]) modeling the times of outcome presentation; 2 regressors modeling button presses across all stimuli: one modeling all "accept" trials and one modeling all "reject" trials; 6 motion parameters as nuisance regressors; bias term modeling the mean activity in each block. Only the 2 regressors modeling the presentation of the two related stimuli (A&B) in each block were used in the "relational structure representation" analysis (as stimulus C was not part of a relational structure, Figure 3 top and Figure S3), while all stimulus presentation regressors (A, B & C) were used in the "visual stimulus identity representation" analysis (Figure 3, bottom). Each condition (a particular stimulus under a particular structure) had two patterns (100-long vectors of spatially pre-whitened regression coefficients) – one from each independent run. We defined the cross-run correlation distance between each pair of conditions by averaging the following quantities: [correlation distance between condition *i* pattern from run 1 and condition *j* pattern from run 2] and [correlation distance between condition *i* pattern from run 2 and condition *j* pattern from run 1]. This resulted in [number of conditions] × [number of conditions] data RDM for each searchlight (8×8 for the "relational structure representation" analysis; 12×12 for the "visual stimulus identity representation" analysis). We then defined a hypothesis-driven contrast between RDM elements: In the main "relational structure representation" analysis, "different structure" elements should be more dissimilar to each other than "same structure" elements. For the control analyses in Figure S3, we ignored elements of the same visual stimulus (Figure S3C) or the same stimuli set (Figure S3D). In the "visual stimulus identity representation" analysis, "different visual stimulus" elements should be more dissimilar than "same visual stimulus" elements. Using the maximum cluster mass statistic (Nichols and Holmes, 2002) for multiple comparisons correction, we report clusters that survived FWE correction at a cluster-forming threshold of $p < 0.001$ (green clusters in Figures 3 and S3).

In addition, to test for a relational structure representation we conducted a similar GLM (GLM2a) where we collapsed the two related stimuli onto the same regressor (Figures 3F and S3B). GLM2a included the following regressors: 2 main effect regressors ([AB],[C]) modeling stimulus presentation times; 2 main effect regressors ([AB],[C]) modeling outcome times; 2 regressors modeling button presses across all stimuli: one modeling all "accept" trials and one modeling all "reject" trials; 6 motion parameters as nuisance regressors; bias term modeling the mean activity in each block. We followed the same protocol as above to construct data RDMs (using the [AB] > baseline contrast, resulting in 4×4 data RDMs) and for statistical inference.

To test whether standard prediction error signals depended on the relational structure (prediction error × structure interaction analysis, Figures 4 and S4), we first wanted to replicate the previously described ventral striatum (vStr) and vmPFC univariate prediction error signals (Delgado et al., 2000; Hampton et al., 2006; O'Doherty et al., 2003; Ramnani et al., 2004). We conducted a GLM (GLM3) which included the following regressors per each block: 2 main effect regressors ([AB],[C]) modeling outcome times; one parametric regressor of the "correctness" prediction error from the STRUCT model, locked to the time of [AB] outcome presentation; 2 main effect regressors ([AB],[C]) modeling stimulus presentation times; 2 regressors modeling button presses across all stimuli: one modeling all "accept" trials and one modeling all "reject" trials; 6 motion parameters as nuisance regressors; bias term modeling the mean activity in each block. For each subject, we calculated the contrast [AB STRUCT "correctness" prediction error] > [baseline], smoothed the contrast image using a 5mm FWHM kernel, and obtained group-level results using SPM (Figures 4B and 4C, insets). As expected, the two strongest peaks were in vStr and vmPFC. We then used these peaks as (unbiased) ROIs for the multivariate prediction error X structure interaction analysis. To this end, in addition to the surface-based cortical searchlight procedure described above, we also defined 100 voxels long volumetric searchlights within an anatomical mask of the vStr (Harvard-Oxford Subcortical Structure Atlas). Unlike the surface-based searchlight, the analyses for the volumetric searchlight were all performed in MNI152 space. Only the regressor modeling the [AB] "correctness" prediction error in each block was used in the RSA analyses for Figures 4 and S4, resulting in 8 patterns – one pattern per block. We again used the cross-run correlation distance to collapse patterns of the same conditions (a particular stimuli set under a particular structure) across runs, this time to construct 4×4 data RDMs – one condition per block type. Again, we defined a hypothesis-driven contrast between RDM elements, where "different structure" elements should be more dissimilar to each than "same structure" elements. We report the (uncorrected) p values obtained by permutation tests in the searchlights centered on the vStr and vmPFC peaks of the univariate prediction error effect (Figure 4). We also present the exploratory, uncorrected results across cortex of this effect (Figure S4C).

Multiple comparisons correction

Multiple comparisons correction was performed using the permutation tests machinery (Nichols and Holmes, 2002) in PALM (Winkler et al., 2014): we first thresholded the (uncorrected) P-map at $p < 0.001$, and measured the mass of all surviving clusters. We then

CellPress
OPEN ACCESS

repeated this procedure for each of the 10000 random sign-flip iterations described above, and created a null distribution of cluster masses by saving only the mass of the largest cluster in each iteration. Comparing the true cluster masses to the resulting null distributions results in FWE-corrected P values.

Leave-one-out analysis - EC effect

To demonstrate the robustness of the EC effect, we ran the pipeline described above for the relational structure effect 28 times, each time leaving out the data from one subject. This resulted in 28 cluster mass FWE-corrected P values within a hemisphere. A histogram of these P values is shown in Figure S3A.

ADDITIONAL RESOURCES

The univariate “correctness” prediction error signal

In Figure 2D, we showed that at stimulus presentation time activity in several brain regions covaries with the value of the chosen option as predicted by the STRUCT model. Importantly, this survives the inclusion of NAÏVE chosen value as a co-regressor. This means the portion of STRUCT value that is *orthogonal* to the NAÏVE value is reflected in fMRI signal even though the two values are correlated ($r = 0.77$). It would have made sense to explore a prediction error-equivalent of this analysis at outcome time. However, this test is a much harder test than the one we performed for value at stimulus presentation time. This can be superficially explained by the fact that STRUCT and NAÏVE prediction errors are much more correlated ($r = 0.93$) than the STRUCT and NAIVE chosen action values ($r = 0.77$), and are hence more difficult to tease apart. In this section we explain in detail the reasons why this test does not work.

Prediction error (PE) is defined as [reward] – [expected value]. In our case, the “correctness” PE is defined as [correctness] – [expected value of the chosen action]. This is exactly the same as the classic reward prediction error (RPE), if an assumption is made that avoiding punishment (correct reject trial) and gaining reward (correct accept trial) are equivalent. For consistency with the classic RPE literature, in the rest of this section we refer to the “correct” signal as “reward,” to “correctness PE” as just “PE,” and to the “expected value of the chosen action” as just “value.”

The definition above implies that a brain region signaling PE should have a positive effect of reward and a negative effect of value at outcome time. If using BOLD signals, these effects should be delayed ~6 s after outcome time – the hemodynamic lag. This is indeed the case in both vStr and vmPFC:

In vStr, these effects can clearly be seen both when STRUCT value and reward are used as co-regressors (Figure S2C, top right) and when NAÏVE value and reward are used as co-regressors (Figure S2C, middle right). The negative effect of value is stronger for STRUCT value than NAÏVE value, perhaps reflecting the existence of structural information in value signals used by vStr.

In vmPFC, while a positive effect of reward at outcome time can be clearly seen (Figure S2C, left plots; see below for a discussion of the seemingly long hemodynamic lag in vmPFC), the effects of value are more complex: both STRUCT value (Figure S2C, top left) and NAÏVE value (Figure S2C, middle left) show a sustained value effect during the period between stimulus presentation (~6.5-8.5 s before outcome) and outcome time (or rather HRF-lag after this period). It is likely that the tail of the HRF of these sustained positive effects interacts with negative effects from outcome time expected from a PE signal, resulting in a response that looks less negative than is vStr.

So far in this section we saw that the predicted PE signal exists in both vStr and vmPFC in GLMs where estimates from STRUCT and NAÏVE models *do not compete* with each other as co-regressors, with stronger effects observed for STRUCT than NAÏVE values. However, we are interested in a stronger test for the PE estimates: a test where both STRUCT and NAÏVE value estimates (as well as reward) are entered as co-regressors in the same GLM. A reasonable prediction in such a GLM would be that if brain regions that encode PE like vmPFC and vStr use structural information to calculate PE, they will still show the PE-signatures of positive reward and negative value effects, but only for STRUCT value. For this to be true, the brain activity at outcome time should reflect the portion of STRUCT value that is *orthogonal* to NAÏVE value, but critically also to reward. A similar test for value estimates at stimulus presentation time in a GLM that included both STRUCT and NAÏVE values yielded strong effects in a variety of brain regions (main text and Figure 2D), including vmPFC (Figure S2C, bottom left, period before outcome). Crucially, at stimulus presentation time there is no reward signal, and so the relevant portion of STRUCT value needs only to be orthogonal to NAÏVE value.

This additional constraint turns out to be critical. The component of STRUCT value that is orthogonal to NAÏVE value is itself correlated with reward ($r = 0.38$). This is not true the other way around: The component of NAÏVE value that is orthogonal to STRUCT value is not correlated with reward ($r = -0.05$). This is to be expected because STRUCT is a better model of the world than NAIVE, so better predicts reward. Hence, any comparison between the STRUCT and NAÏVE value effects in the presence of a reward regressor is not a fair one. The strong reward effect observed in both vStr (Figure S2C bottom right) and vmPFC (Figure S2c bottom left) implies that brain activity in these areas covaried with the component of reward that was orthogonal to both STRUCT and NAÏVE value regressors.

In summary, a large portion of the fMRI signal in vStr and vmPFC is correlated with reward. The remaining portion *is* correlated negatively with value (as expected from a PE signal), but to determine which value we need to project the variance into a space that is *itself* correlated with reward.

Characteristics of the HRF in vmPFC

The peri-stimulus plots in Figure S2C reveal an interesting phenomenon: the vmPFC prediction error signal (positive effect of reward and negative effect of value) peaks 8.5 s after outcome presentation. This is in contrast to the PE signal in the vStr, which peaks after 6 s – the default value widely used in fMRI processing software. This suggests that the delay of the hemodynamic response function (HRF) in the vmPFC is longer than in the HRF it is usually modeled with. To our knowledge this has never been systematically investigated and reported, but anecdotal observations from our lab are consistent with this finding.

We repeated the prediction error × structure interaction analysis from Figure 4 using the SPM HRF with the delay period changed from 6 to 8.5 s. The univariate PE effect in the vmPFC was indeed much stronger when using the delayed HRF (peaks: 8.5 s HRF $t(27) = 16.63$, MNI [-4,52,-6]; 6 s HRF $t(27) = 9.3$, MNI [-2,48,-18]). Importantly, the PE × relational structure effects at these univariate vmPFC peaks were significant also when conducting the entire analysis with the delayed HRF (left hemisphere univariate peak (MNI [-4,52,-6]): $p = 0.02$; right hemisphere univariate peak (MNI [2,46,-10]): $p = 0.04$).

Neuron, Volume *109*

Supplemental Information

Entorhinal and ventromedial prefrontal cortices abstract and generalize the structure of reinforcement learning problems

Alon Boaz Baram, Timothy Howard Muller, Hamed Nili, Mona Maria Garvert, and Timothy Edward John Behrens

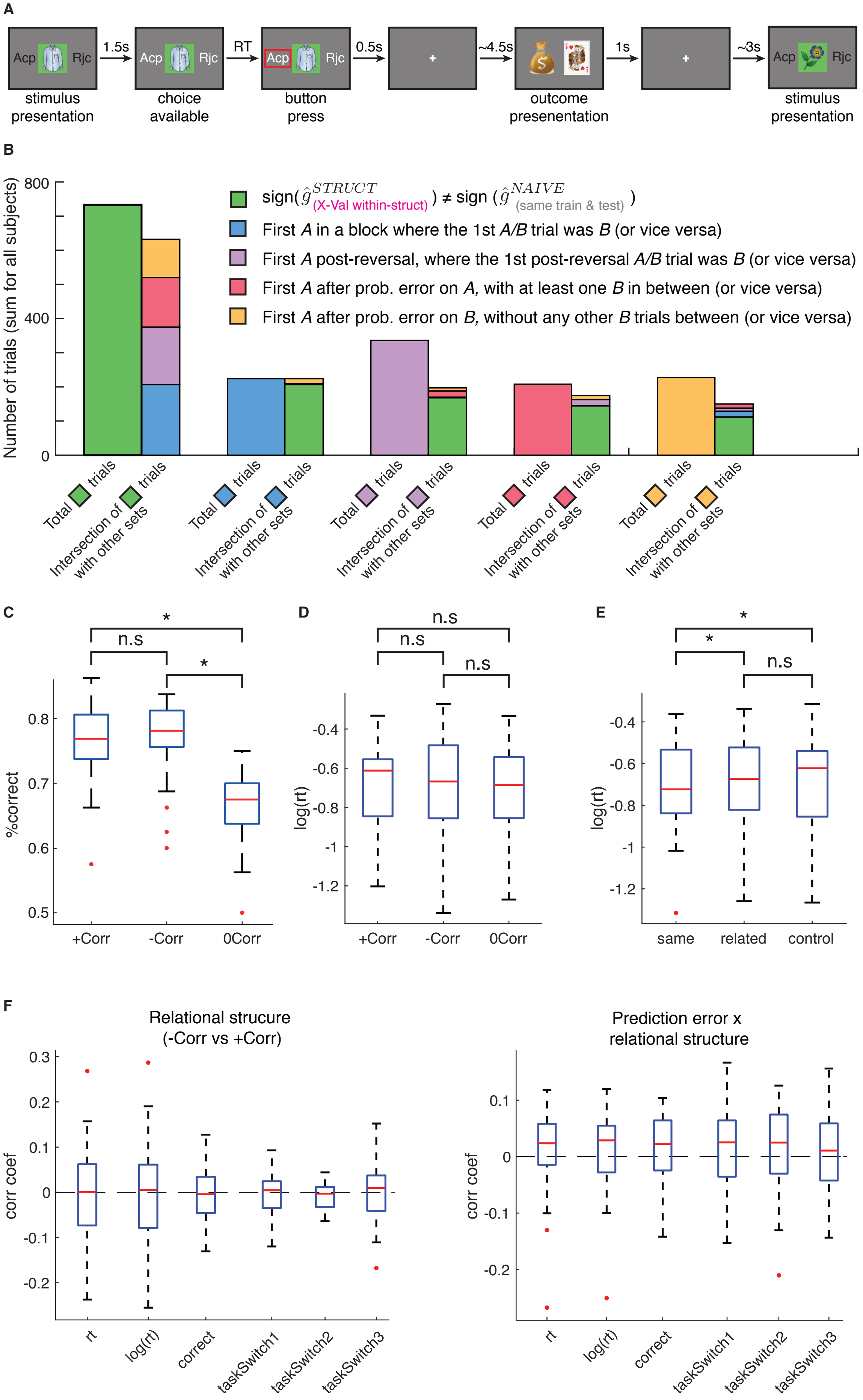
A
Acp
Rjc
1.5s
RT
0.5s
~4.5s
1s
~3s
stimulus presentation
choice available
button press
outcome presenentation
stimulus presentation
B
Number of trials (sum for all subjects)
800
400
0
First A in a block where the 1st A/B trial was B (or vice versa)
First A post-reversal, where the 1st post-reversal A/B trial was B (or vice versa)
First A after prob. error on A, with at least one B in between (or vice versa)
First A after prob. error on B, without any other B trials between (or vice versa)
Total trials
Intersection of trials with other sets
C
%correct
+Corr
-Corr
0Corr
n.s
*
D
log(rt)
E
same
related
control
F
Relational strucure (-Corr vs +Corr)
Prediction error x relational structure
corr coef
rt
log(rt)
correct
taskSwitch1
taskSwitch2
taskSwitch3

Figure S1. A. Time course of an example trial (related to Figure 1)**.** Acp: Accept; Rjc: reject. RT: reaction time. **B-E: Additional behavioural analyses** (related to Figure 2)**. B.** The trials used in Figure 2B are mostly the first trial in a block where the STRUCT model is useful, or trials following probabilistic errors in outcomes or reversal. We identified 4 sets of trials that constituted the majority of the trials where the within-structure cross-validated STRUCT model (pink elements in Figure 2A; Figure 2B, left) and the NAÏVE model trained and tested on the same data (grey elements in Figure 2A; Figure 2B, right) made different predictions. For each set of the 5 sets (the set of trials used in Figure 2B and the 4 sets identified by their schedule properties), we plotted two bars: the overall number of trials of that set across all participants (left, wider bar), and the number of trials at the intersection of that set with other sets (right, narrower bar). The number of trials at the intersection of any 3 sets was negligible (<14). 86% of the trials used in Figure 2B (green, most left bar) are members of at least one other set. **C.** Subjects performance (measured as % of correct choices, according to the ground truth outcome probability) was better in trials of the related stimuli than trials of the control stimulus (two-tailed paired t-tests: +Corr vs 0Corr $t(27)=6.7$, $P<10^{-6}$; -Corr vs 0Corr paired $t(27)=7.11$, $P<10^{-6}$, -Corr vs +Corr paired $t(27)=0.43$ $P=0.66$). **D.** log(reaction times), split by correlation type of trial (-Corr, +Corr or control). Reaction times did not differ between trials of stimuli under different types of correlations (two-tailed paired t-tests: $P>0.4$ for all comparisons). **E.** Task switching effects. We split all trials to three groups according to the relationship between current and previous trial: "same" (A→A (A trials preceded by an A trial), B→B, C→C), "related" (A→B, B→A) and "control" (A→C, B→C, C→A, C→B), and compared the means of log(reaction times) of these groups across subjects. As expected, subjects were quicker to respond to stimuli preceded by the same stimulus (one-tailed paired t-test on log(reaction times): same vs related: $t(27)=2.1$, $P=0.02$, same vs control: $t(27)=2.88$, $P=0.003$). However, there was no significant difference between log(reaction times) in trials of stimuli presented after their related stimulus ("related") compared to trials where stimuli were presented after an unrelated stimulus ("control"), though a weak trend in the expected direction was observed (related vs control: $t(27)=0.41$, $P=0.34$). **F. Possible confounds** (related to figures 3 and 4). Correlation coefficients of possible behavioural confounds with the effects of interest. We first constructed six confound regressors: *reaction time*, *log(reaction time)*, *correct* (-1/1 for trials where the subject's choice was incorrect/correct according to the ground truth outcome probability, respectively), and three task switching regressors where we partitioned the three "task switching groups" (same, related, control – see panel **E**) in different ways, reflecting possible levels of task switching: *taskSwitch1* ("same": -1, "related": 0, "control": 1), *taskSwitch2* ("same" and "related": -1, "control": 1) and *taskSwitch3* ("same": -1, "related" and "control": 1). Next we constructed regressors reflecting our effects of interest: *relational structure* (-Corr: -1, +Corr: 1, control trials were ignored) and *correctness prediction error x relational structure interaction*. Both regressors showed no significant correlations with any of the confound regressors. In all plots, the red central line is the median, the box edges are the 25th and 75th percentiles, the whiskers extend to the most extreme data points that are not considered outliers, and the outliers are plotted as red circles.

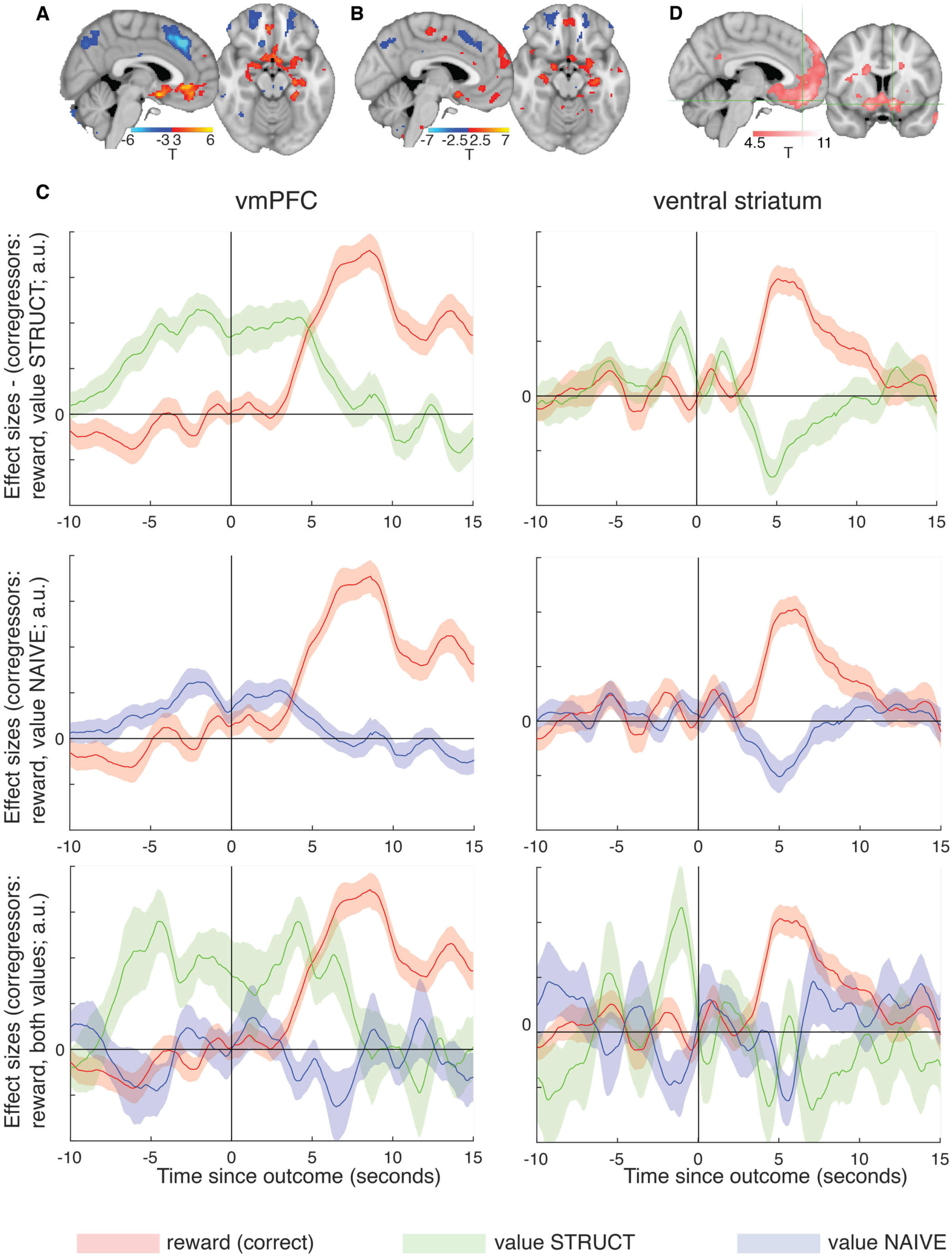

Figure S2. Control analyses for the univariate contrasts (related to "*Additional resources*" section in *STAR methods*, and Figures 2D and 4). **A.** Effect of the contrast [STRUCT chosen action value] > [baseline], locked to stimulus presentation time (same contrast as is Figure 2D), in a GLM that is similar to GLM1 but includes reaction time as a covariate. **B.** Effect of the contrast [STRUCT chosen action value] > [NAÏVE chosen action value], locked to stimulus presentation time, from the same GLM as in Figure 2D (GLM1; note the lower

threshold – no effects survived multiple comparisons correction for this contrast). **C.** Peri-stimulus plots showing the vmPFC (left) and vStr (right) effect time courses of the reward, STRUCT value and NAÏVE value locked to outcome presentation. Time courses are not corrected for the hemodynamic lag. Top: GLM including reward ("correct") and STRUCT chosen action value as co-regressors. Middle: GLM including reward and NAÏVE chosen action value. Bottom: GLM including reward and *both* STRUCT and NAÏVE chosen action values. Prediction error signals (positive reward and negative value effects) can be clearly seen in vStr when the two values do not compete in the same GLM (top and middle right panels). This is also true in vmPFC, once accounting for the sustained value effect from stimulus presentation time (~6.5-8.5 seconds before outcome time) to outcome time (top and middle left panels). However, while the reward effects are still present in a GLM that includes both STRUCT and NAÏVE values, there are no negative value effects due to collinearities between regressors (bottom panels). Data taken from the peaks of the vStr and vmPFC peaks of the STRUCT prediction error effect, used for the prediction error x relational structure interaction effect (Figure 4). **D.** Effect of the contrast [STRUCT correctness prediction error] > [baseline], locked to outcome presentation time (same contrasts as the insets in Figure 4B and 4C), in a GLM that is similar to GLM3 but includes reaction time as a covariate.

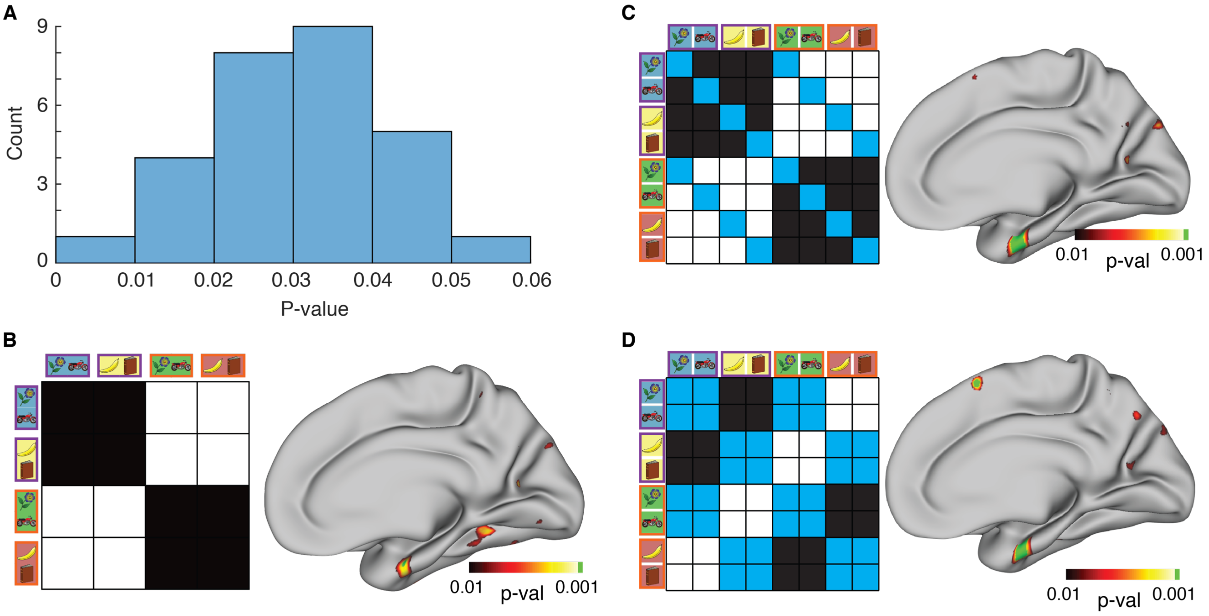

Figure S3. Control contrasts for the EC relational structure effect (related to Figure 3, top)**. A.** Histogram of the leave-one-subject-out cluster-mass FWE-corrected P-values of the EC relational structure effect. **B-D**: Left: model RDMs. Black elements should be similar, white elements should be dissimilar, blue elements are ignored. Right: whole-surface results (right hemisphere). Colour map is the same as in **Figure 3**. B. Relational structure effect from a GLM where the related stimuli (A and B) are collapsed onto the same regressor (GLM2a). **C.** Relational structure effect (GLM2, ignoring all elements of pairs of same-stimulus conditions. **D.** Relational structure effect (GLM2), ignoring all elements of pairs of same stimuli set conditions.

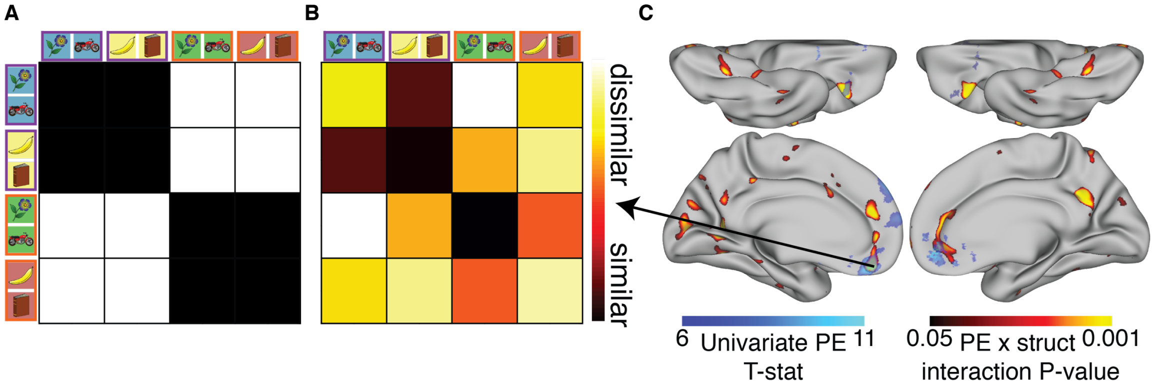

Figure S4. Prediction error x relational structure interaction effect (related to Figure 4)**. A.** Model RDM. Note that each pair of related stimuli correspond to only one condition in the RDM as the trials of each pair are collapsed in GLM3. **B.** Visualisation of the data RDM of the prediction error x relational structure interaction effect at the peak of the vmPFC univariate prediction error effect (MNI [-4,44,-20]). **C.** Visualisation of whole-surface results of the multivariate prediction error x relational structure interaction effect (red-yellow, effects do not survive FWE-correction across a cortical hemisphere - these are exploratory results; note the low threshold), overlaid on the univariate prediction error effect, used to define the ROIs (blue – same as in the insets of Figure 4B and 4C but with a higher threshold). We found bilateral effects in vmPFC (peak P-values: LH: P=0.002 uncorrected, Figure 4A,B; RH: P=0.01 uncorrected, Figure 4C), in close proximity to each hemisphere's univariate peak. The strongest effects in cortex where observed in PCC RH (right hemisphere): P=0.001 uncorrected) and vlPFC (LH: P < 0.001 uncorrected, P=0.002 at the peak of the univariate prediction error vlPFC effect). In all of these regions, the effects were also observed bilaterally, albeit weaker (PCC LH: P = 0.005 uncorrected; vlPFC RH: P=0.005 uncorrected).

Day	Sess	Stimuli Sets per block (each number a single stimuli set - a unique triplet of images. Triplets sampled independently for each subject)	Structure Per block (correlation between outcome probabilities of two stimuli, order counterbalanced across subjects)	Stimuli background color (Blue, Green, Red, Yellow, Counterbalanced across subjects)	Trials per block (same for all subjects)	Feedback (FB, same for all subjects)
1	1	5,5,5,5,5,5,5,5	-,-,-,-,-,-,-,-	None	60	Full
1	2	6,6,6,6,6,6,6,6	-,-,-,-,-,-,-,-	None	60	Full
2	1	7,7,7,7,7,7,7,7	+,+,+,+,+,+,+,+	None	60	Full
2	2	8,8,8,8,8,8,8,8	+,+,+,+,+,+,+,+	None	60	Full
3	1	1,1,1,1,1,1,1,1	-,+,-,+,-,+,-,+	B,G,B,G,B,G,B,G	60	No FB on reject trials in last 15 trials
3	2	2,2,2,2,2,2,2,2	+,-,+,-,+,-,+,-	R,Y,R,Y,R,Y,R,Y	60	No FB on reject trials in last 15 trials
4	1	1,1,2,2,1,1,2,2	-,+,-,+,+,-,+,-	B,G,Y,R,G,B,R,Y	60	No FB on reject trials in last 15 trials
Pre-scan	1	2,2,1,1	-,+,-,+	Y,R,B,G	60	Full
scan	1	1,2,1,2,1,1,2,2	-,+,+,-,-,+,-,+	B,R,G,Y,B,G,Y,R	30	Full

Table S1. Training schedule for an example subject (Related to "*Method detail*" in *STAR Methods*).

2. Model	# parameters	Log likelihood	AIC	BIC
NAÏVE	2 params x<br>2 structures x 28 subjects = 112	-4515.36	9254.72	10077.2
STRUCT	5 params x<br>2 structures x 28 subjects = 280	-2894.44	6348.89	8405.06

Table S2. Formal model comparison between STRUCT and NAÏVE models (related to Figure 2 and to "*Quantification and Statistical Analysis*" section in *STAR methods*). NAIVE parameters: learning rate and inverse temperature. STRUCT parameters: learning rate, inverse temperature and 3 cross-terms.

Peak MNI coords	Brain region(s) of cluster	Peak t-score (df=27)	p-value (FWE-corrected on cluster level)
-32,-16,-20	L anterior hippocampus + amygdala + entorhinal cortex	6.71	P<0.001
-4,10,-12	Posterior mPFC (subcallosal cortex)	6.45	P=0.001
-6,58,36	Dorsal mPFC	4.56	P<0.001
28,-44,64	L Somatosensory cortex	5.56	P=0.006
0,34,-8	vmPFC	5.53	P<0.001
-32,-20,2	Insula/parietal operculum/white matter	5.07	P<0.001
26,4,-22	R hippocampus + amygdala + entorhinal cortex	5	P=0.003
52,-32,20	R parietal operculum	4.88	P<0.001
8,34,42	ACC	6.92	P<0.001
-42,16,6	L Insula	6.68	P<0.001
46,-52,34	R Angular gyrus	6.03	P<0.001
40,44,-2	OFC	5.69	P=0.002

Table S3. Effect of chosen action value of STRUCT model, when competing with NAÏVE model in the same GLM (GLM1, related to Figure 2D). The contrast is [STRUCT chosen action value] > Baseline. All clusters with a FEW-corrected P-value < 0.05 are reported. Note that negative effects (with a negative t-score) are also reported.
